# Lipid Nanoparticle-Mediated Hit-and-Run Approaches Yield Efficient and Safe *In Situ* Gene Editing in Human Skin

**DOI:** 10.1021/acsnano.3c08644

**Published:** 2023-11-02

**Authors:** Juliana Bolsoni, Danny Liu, Fatemeh Mohabatpour, Ronja Ebner, Gaurav Sadhnani, Belal Tafech, Jerry Leung, Selina Shanta, Kevin An, Tessa Morin, Yihang Chen, Alfonso Arguello, Keith Choate, Eric Jan, Colin J.D. Ross, Davide Brambilla, Dominik Witzigmann, Jayesh Kulkarni, Pieter R. Cullis, Sarah Hedtrich

**Affiliations:** † Faculty of Pharmaceutical Sciences, University of British Columbia, 2405 Wesbrook Mall, Vancouver V6T 1Z3, BC, Canada; ‡ Berlin Institute of Health @ Charité Universitätsmedizin, Berlin 10117, Germany; § Department of Biochemistry and Molecular Biology, University of British Columbia, 2350 Health Sciences Mall, Vancouver V6T 1Z3, BC, Canada; ∥ NanoVation Therapeutics, 2405 Wesbrook Mall, Vancouver V6T 1Z3, BC, Canada; ⊥ University of Montréal, Faculty of Pharmacy, Montréal H3T 1J4, Quebec, Canada; # Departments of Dermatology, Genetics, and Pathology, Yale University School of Medicine, New Haven 06510, Connecticut, United States; ∇ Department of Infectious Diseases and Respiratory Medicine, Charité - Universitätsmedizin Berlin, corporate member of Freie Universität Berlin and Humboldt Universität, Berlin 10117, Germany; ○ Max-Delbrück Center for Molecular Medicine in the Helmholtz Association (MDC), Berlin 13125, Germany

**Keywords:** lipid nanoparticles, gene delivery, gene editing, skin, ARCI, genodermatoses, base editing

## Abstract

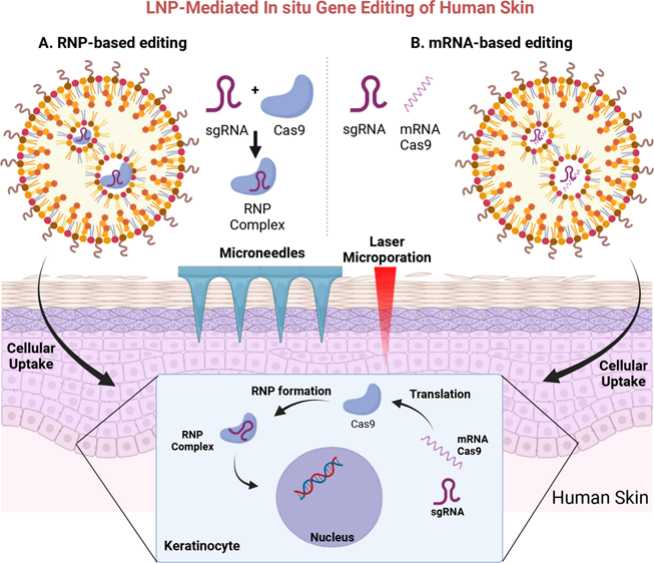

Despite exciting advances in gene editing, the efficient delivery of genetic tools to extrahepatic tissues remains challenging. This holds particularly true for the skin, which poses a highly restrictive delivery barrier. In this study, we ran a head-to-head comparison between Cas9 mRNA or ribonucleoprotein (RNP)-loaded lipid nanoparticles (LNPs) to deliver gene editing tools into epidermal layers of human skin, aiming for *in situ* gene editing. We observed distinct LNP composition and cell-specific effects such as an extended presence of RNP in slow-cycling epithelial cells for up to 72 h. While obtaining similar gene editing rates using Cas9 RNP and mRNA with MC3-based LNPs (10–16%), mRNA-loaded LNPs proved to be more cytotoxic. Interestingly, ionizable lipids with a p*K*_a_ ∼ 7.1 yielded superior gene editing rates (55%–72%) in two-dimensional (2D) epithelial cells while no single guide RNA-dependent off-target effects were detectable. Unexpectedly, these high 2D editing efficacies did not translate to actual skin tissue where overall gene editing rates between 5%–12% were achieved after a single application and irrespective of the LNP composition. Finally, we successfully base-corrected a disease-causing mutation with an efficacy of ∼5% in autosomal recessive congenital ichthyosis patient cells, showcasing the potential of this strategy for the treatment of monogenic skin diseases. Taken together, this study demonstrates the feasibility of an *in situ* correction of disease-causing mutations in the skin that could provide effective treatment and potentially even a cure for rare, monogenic, and common skin diseases.

Exciting and fast-paced advances in the field of clustered regularly interspaced short palindromic repeats (CRISPR)/Cas9-mediated gene editing now provide us with powerful tools to manage previously untreatable conditions.^1^ Since its discovery in 2012, CRISPR-based programmable gene editing has evolved rapidly. As such, clinical trials and gene editing data from patients suffering from sickle cell anemia or beta-thalassemia have already showcased its potential.^2^ As of today, we can theoretically correct >90% of disease-causing mutations using increasingly precise gene editing tools such as base or prime editors.^3^ These techniques are particularly promising for orphan diseases that often lack effective treatment options and have a very high unmet clinical need.^4^

While there is a strong interest in developing efficient gene editing and delivery strategies for tissues such as the liver,^5,6^ the eyes,^7^ and muscles,^8^ other organs including the skin have received little attention so far. The skin, however, is our largest organ, and its barrier properties are critical for our survival. Also, skin diseases significantly impact our physical and psychological well-being.^9^ This applies to common diseases such as atopic dermatitis but even more so to genodermatoses, a diverse group of rare, often highly stigmatizing diseases. Genodermatoses result from single mutations in ≤500 different genes and have a dramatic impact on patient quality of life and life expectancy in certain cases.^10−12^ One example is autosomal, recessive, congenital ichthyosis (ARCI), which refers to a heterogeneous group of severe keratinization disorders.^13,14^ While the severity may vary, the symptoms are especially significant in neonates that may suffer from higher mortality rates due to increased transepidermal water loss or infections.^11,15,16^ Targeted treatment options are currently not available.^12^

A major challenge that still prevents us from unlocking the full potential of gene editing is the lack of efficient and safe delivery strategies to the target cells and tissue.^8^ This holds especially true for the skin, which forms a very tight barrier even in some diseased states. When targeting the skin, an intravenous application will most likely not yield an efficient delivery of genetic cargo. The lack of vasculature in the viable epidermis (the target for most genodermatoses) and the tight epidermal–dermal junction zone prevent the delivery of nucleic acid payloads. Hence, enabling gene editing for skin diseases requires either treatment of cells outside of the human body (*ex vivo* approach) followed by regrafting or a topical application (*in situ* approach).^12^ The latter is challenging due to the barrier properties of the skin and the unfavorable characteristics of genetic cargo such as their high molecular weight, negative charge, and instabilities.

While the adeno-associated virus (AAV) or lentivirus (LV) are potent *in vivo* gene delivery vectors,^17^ their application is limited by safety concerns (e.g., immuno- and mutagenicity), high production costs, and packaging constraints (4.7 kilobases for AAVs and 10 kb for LV). The latter is particularly challenging when delivering gene editing tools like CRISPR/Cas, a 2- to 3-component system depending on the editing approach.^18^ In fact, it requires multiple viruses to deliver the ribonucleoprotein (RNP) complexes (single guide (sg) RNA and Cas9 protein) and donor templates.^19^ The packaging constraints are even more pronounced for base and prime editors.^20,21^

Hence, there is a great need for alternative, nonviral delivery systems. Lipid nanoparticles (LNPs) are the most advanced nonviral delivery systems to date.^22−24^ A large body of evidence demonstrates their safety and efficacy since the approval of Onpattro, an siRNA-based drug, and more recently the COVID-19 mRNA vaccines.^25,26^ These success stories have sparked an unparalleled interest in LNPs for gene delivery.^27^

In this study, we investigated the potential of LNPs to deliver gene editing tools into the viable epidermis of human skin with the long-term goal to enable efficient *in situ* correction of disease-causing mutations, which could provide an effective treatment and potentially a cure for rare, monogenic skin diseases. We compared the suitability of LNPs for Cas9 mRNA and ribonucleoprotein (RNP) delivery into primary human skin cells and tissue and the impact of LNP composition on skin transfection and editing rates, assessed sgRNA-dependent off-target effects by rhAMP sequencing, and ultimately demonstrated the correction of a disease-causing mutation in ARCI patient cells using lipid-based transfection. We were particularly interested in a head-to-head comparison between Cas9 mRNA and RNP-based gene editing and assessed the versatility of LNPs for RNP delivery in this context.

As such, this study demonstrates the feasibility of *in situ* gene editing of human skin, facilitating the development of alternative therapies for rare and common skin diseases.

## Results and Discussion

### LNP Composition and Genetic Payload Determine Gene Editing Efficiency in Primary, Human Keratinocytes

**Figure 1:**
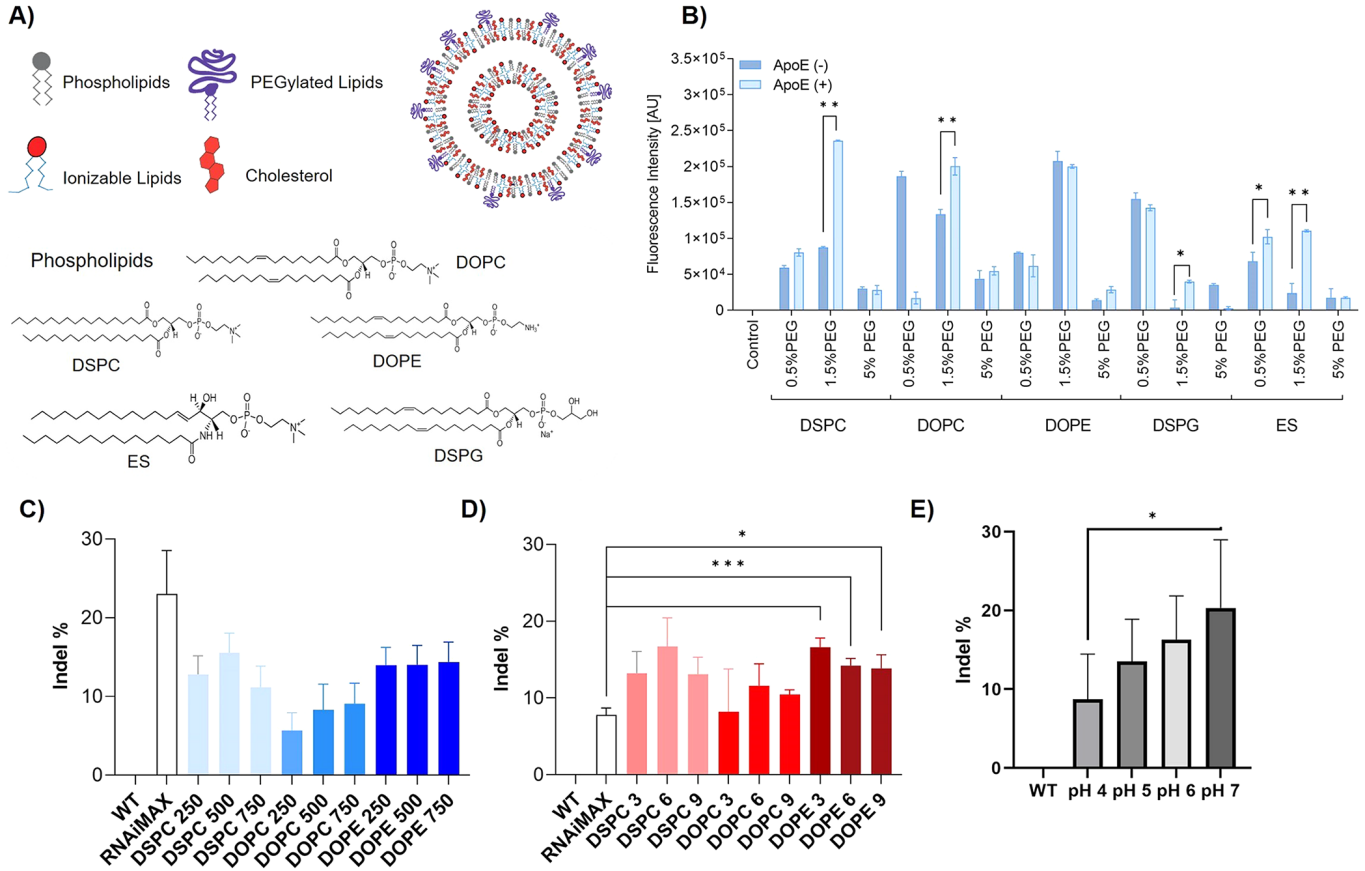
(A) Schematic depiction of LNPs and chemical structures of the helper lipids (DSPC, DOPC, DOPE, ES, DSPG) used for LNP preparation. (B) Cell uptake efficiency of LNPs containing different helper lipids 24 h after incubation with primary keratinocytes (KCs). (C, D) Frequency of indel% (normalized to wild-type (WT) cells) in the model gene *HPRT* after transfection of KCs with (C) RNP- and (D) mRNA-loaded LNPs at three different L/R (mol/mol) and N/P (mol/mol) ratios, respectively. * indicates statistically significant differences over RNAimax; *p < 0.05; **p < 0.01; ***p < 0.001. (E) Effect of RNP-loading at different pH on indel% (normalized to WT) indicative of gene editing efficacies in the model gene *HPRT*. Data are presented as the mean ± SD of at least three biologically independent replicates.

It is well-established that the LNP composition significantly affects cell uptake^28^ and transfection efficacies.^29,30^ Hence, we first screened a pool of LNP formulations to identify compositions that are efficiently taken up by the target cells, primary human keratinocytes (KCs), that constitute ≥90% of the epidermal skin layer. Therefore, we assessed the internalization of LNP composed of five different helper lipids that vary structurally and charge-wise (Figure 1A; DOPC, DOPE, DSPC, ES, and DSPG). Lipid charges can dramatically affect the delivery efficiency by altering the physiochemical properties of the LNP, such as p*K*_a_ and surface charge. This in turn can affect the protein corona formation and receptor-mediated endocytosis, thereby impacting LNP uptake.^23,31−33^ Simultaneously, we tested the impact of increasing poly(ethylene glycol) (PEG) concentrations (0.5%, 1.5%, and 5%) and cell uptake in the presence and absence of apolipoprotein (Apo) E, which facilitates cell uptake via the low-density lipoprotein receptor *in vitro* and *in vivo*.^34,35^

Cell internalization varied significantly (Figure 1B), which is in line with previous work demonstrating the role of helper lipids on cellular uptake.^35^ ApoE improved the LNP cell internalization for most helper lipids, although the impact varied. Except for DSPG LNPs, 1.5% PEGylated LNPs consistently yielded the highest uptake rates. 5% PEG decreased the cell internalization, which is also in line with previous studies.^36^ Based on these results, we decided to proceed with ApoE addition and 1.5% PEGylated DSPC-, DOPE, and DOPC-LNPs.

We next loaded LNPs with Cas9 RNP (1:1 weight ratio Cas9 protein/sgRNA) or Cas9 mRNA (1:1 ratio mRNA/sgRNA) allowing a head-to-head comparison of both Cas9 formats. As the standard LNP microfluidic mixing and loading could not be employed as the ethanol and shear forces would denaturate the RNP,^37,38^ we opted for a benchtop mixing approach where Cas9 mRNA and RNP were added to preformulated, empty LNPs. While this resulted in lower mRNA and RNP encapsulation efficiencies compared to conventional microfluidic mixing (18% for RNP and 31% for mRNA versus 93% mRNA with microfluidic mixing (Figure S1iv)), this proved sufficient for initial biological screening as previously demonstrated for siRNA.^39^ Notably, higher RNP loading efficiencies (up to 64%) were reported for LNP formulations containing permanently cationic lipids.^40^

While mRNA loading did not significantly affect the LNP size, it increased from 25 to 36 nm to 200–280 nm after encapsulation of RNP complexes (Figure S1), which is in line with other reports.^40^ RNP aggregates under acidic LNP complexation conditions resulting in an increased hydrodynamic diameter (regular 10 nm versus 150 nm).^41,42^ In addition, weaker electrostatic interactions between LNP and RNP compared to those of the strong negatively charged mRNA backbone lead to imperfect RNP encapsulation. This also explains the increased polydispersity of LNP-RNP formulations (polydispersity index (PDI) between 0.4–0.75) compared to unloaded and mRNA-loaded LNPs (0.16–0.29). The zeta potential of the particles was as expected: at pH 4, LNPs exhibited a positive surface charge between +5 and +15 mV due to the charged state of the ionizable lipid, which dropped to values close to zero at physiological pH (Figure S1).

We then quantified the gene editing efficacies of the different LNP formulations in primary human KCs and assessed the impact of different lipid-to-RNP (L/R) (250, 500, 750; for RNP) and nitrogen-to-phosphate ratios (N/P 3, 6, 9; for mRNA) that were selected based on previous work.^36^ Gene editing efficacies were determined by a PrimeTime quantitative polymerase chain reaction (qPCR) assay (Figure S2A) that quantifies the percentage of indel formation (small insertions or deletions of ≤50 base pairs) indicative of gene editing in our exemplary target locus *HPRT* using intercalating dyes. This assay accurately detects the frequency of gene edits at ≥5% (Figure S2B).

In KCs, we observed indels of 5–15% with RNP-loaded LNPs and 10–16% with mRNA-loaded LNPs depending on the ratio and the helper lipid (Figure 1C,D). Although the overall impact of the L/R and N/P ratio was lower than expected, the L/R ratio of 500 and N/P ratio of 6 consistently performed best. Interestingly, adding up to 40% cationic lipid 1,2-dioleoyl-3-trimethylammonium propane (DOTAP) increased the editing rates to ∼20% (Figure S3).

Interestingly, lipofection with RNAimax, which served as a reference, yielded the highest gene editing efficacies with ∼24% for RNP but proved less efficient for mRNA delivery than LNP. This finding may also point toward challenges related to RNP loading in LNP. They are typically loaded in an acidic buffer (pH 4) which ensures that the ionizable lipids are positively charged and the negatively charged genetic cargo can be efficiently encapsulated. As outlined above, pH 4 causes protein denaturation and hence activity loss. Encapsulating RNP at neutral pH is difficult, as MC3 (p*K*_a_ 6.0–6.4), the ionizable cationic lipid used here, is uncharged at neutral pH.^30,43^ To assess the impact of the buffer pH on the RNP activity, we varied the pH during loading and then assessed the editing efficacy. Interestingly, less acidic conditions resulted in significantly higher indel formation indicative of higher RNP activity (Figure 1E). These data also show that the RNP complex tolerates an acidic pH for a short period of time without undergoing complete inactivation.

### mRNA-Loaded LNPs Are More Cytotoxic and Intracellularly Trafficked Differently than RNP-LNP

**Figure 2:**
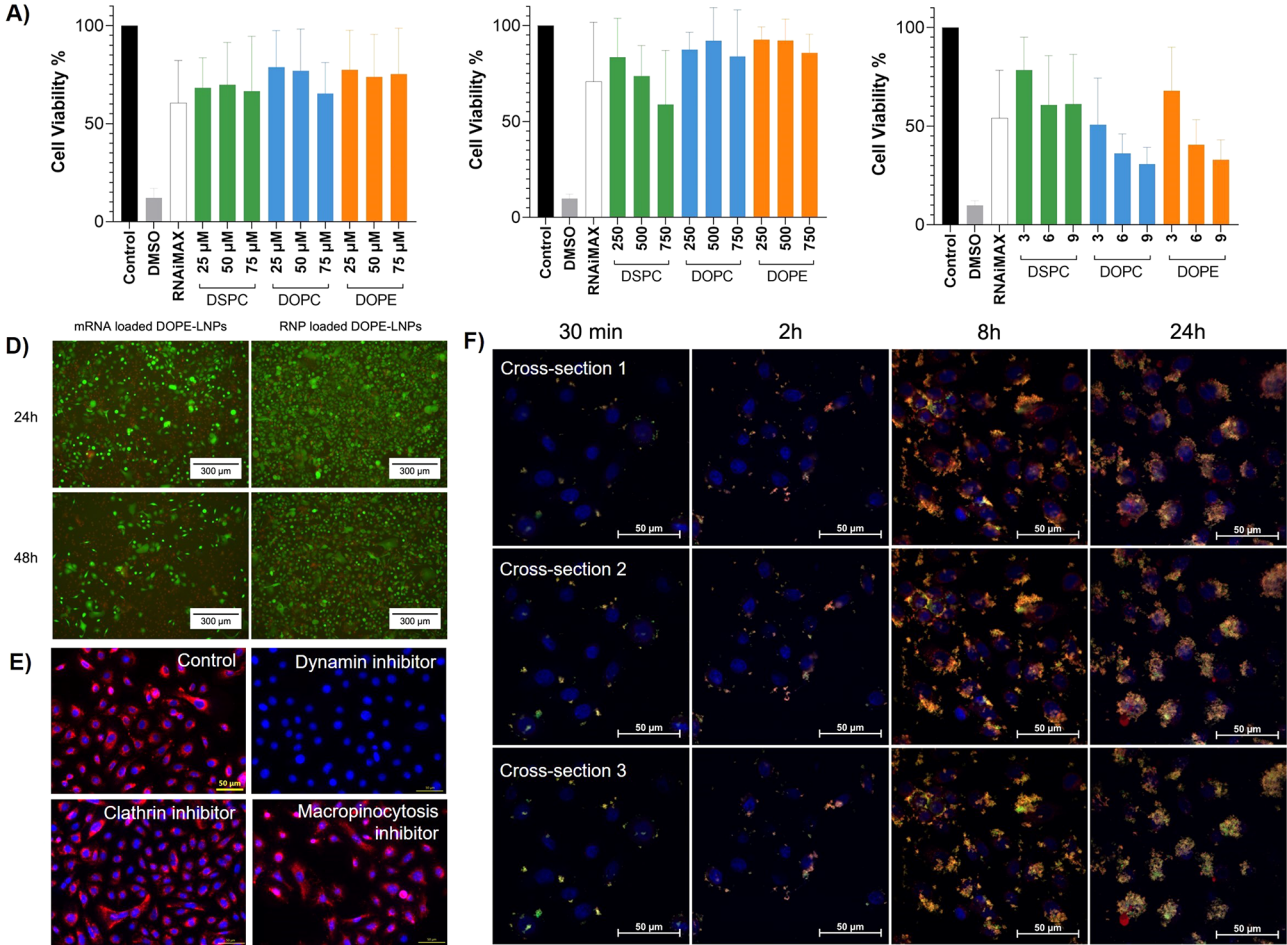
Cell viability of primary human KCs after exposure to (A) unloaded LNPs (μM refers to lipid concentration), (B) RNP-loaded LNPs (depicted as lipid-to-RNP (L/R) ratio), and (C) mRNA-loaded LNPs (depicted as nitrogen-to-phosphate (N/P) ratio) at different concentrations and ratios after 48 h. Data are presented as the mean of three biological replicates ± SD. (D) Representative live/dead cell assay images comparing the toxicity of RNP and mRNA-loaded LNP over 48h. Green is indicative of viable cells; red dots represent dead cells. (E) Preincubation with endocytosis pathway inhibitors indicates that LNP internalization in KCs is mainly dynamin-dependent. Scale bars = 50 μm. (F) Confocal microscopy images showing the time-dependent cell uptake kinetics of RNP-loaded LNPs over 24h.

Next, we determined the cytotoxicity of LNPs in KCs by assessing their metabolic activity via 3-(4,5-dimethylthiazol-2-yl)-2,5-diphenyltetrazolium bromide (MTT) assay and live/dead cell staining. First, the MTT data indicate distinct interindividual sensitivity dependent on the cell donor. Overall, empty and RNP-loaded LNPs resulted in cell viabilities >70%, whereas mRNA-loaded LNPs triggered more pronounced cytotoxicity especially at N/P 6 and N/P 9 (Figure 2A–C). Live/dead cell assays confirmed the MTT data (Figures 2D and S4). This is noteworthy given that the total administered lipid dose is lower for mRNA-loaded compared to unloaded and RNP-loaded LNP: 17, 35, 52 μM total lipids at N/P 3; 6; 9 vs 25, 50; 75 μM total lipid at L/R 250; 500; 750; respectively. This points toward mRNA-specific cytotoxicity, which was corroborated by cell viability assays using increasing mRNA concentrations (Figure S3B). Interestingly, the addition of DOTAP had positive effects on the cell viability overall, maybe due to an increased particle size at higher DOTAP concentrations, although this requires further investigations.

We next determined the cell uptake kinetics and intracellular trafficking of LNPs in KCs. While only little intracellular localization was visible after 30 min and 2 h, significant uptake of both DiI-labeled LNPs and green fluorescent protein (GFP)-tagged RNP occurred after 8h, which further increased over 24h (Figure 2F). The orange color represents RNP/LNP colocalization indicative of concomitant cellular uptake. We next assessed LNP uptake mechanisms in KCs by preincubating the cells with endocytosis pathway inhibitors. No difference in LNP uptake was observed after pretreatment with clathrin- and actin-mediated cell uptake inhibitors. However, a dramatic decline of LNP uptake was observed when blocking dynamin-dependent pathways (Figure 2E) suggesting that LNP uptake in KCs is largely dynamin-dependent.^8^ It is well-established that LNP internalization is cell-type specific and contingent on the LNP composition.^44^ For example, in HeLa, HuH7,^45^ primary human adipocytes,^46^ and hepatocytes,^34^ LNP uptake has been previously linked to low-density lipoprotein (LDL) receptor-mediated endocytosis, which mainly occurs via clathrin-coated vesicles.^47^ Our findings add that alternative dynamin-dependent processes such as caveolae- and endophilin-mediated endocytosis may also contribute to LNP internalization.

**Figure 3:**
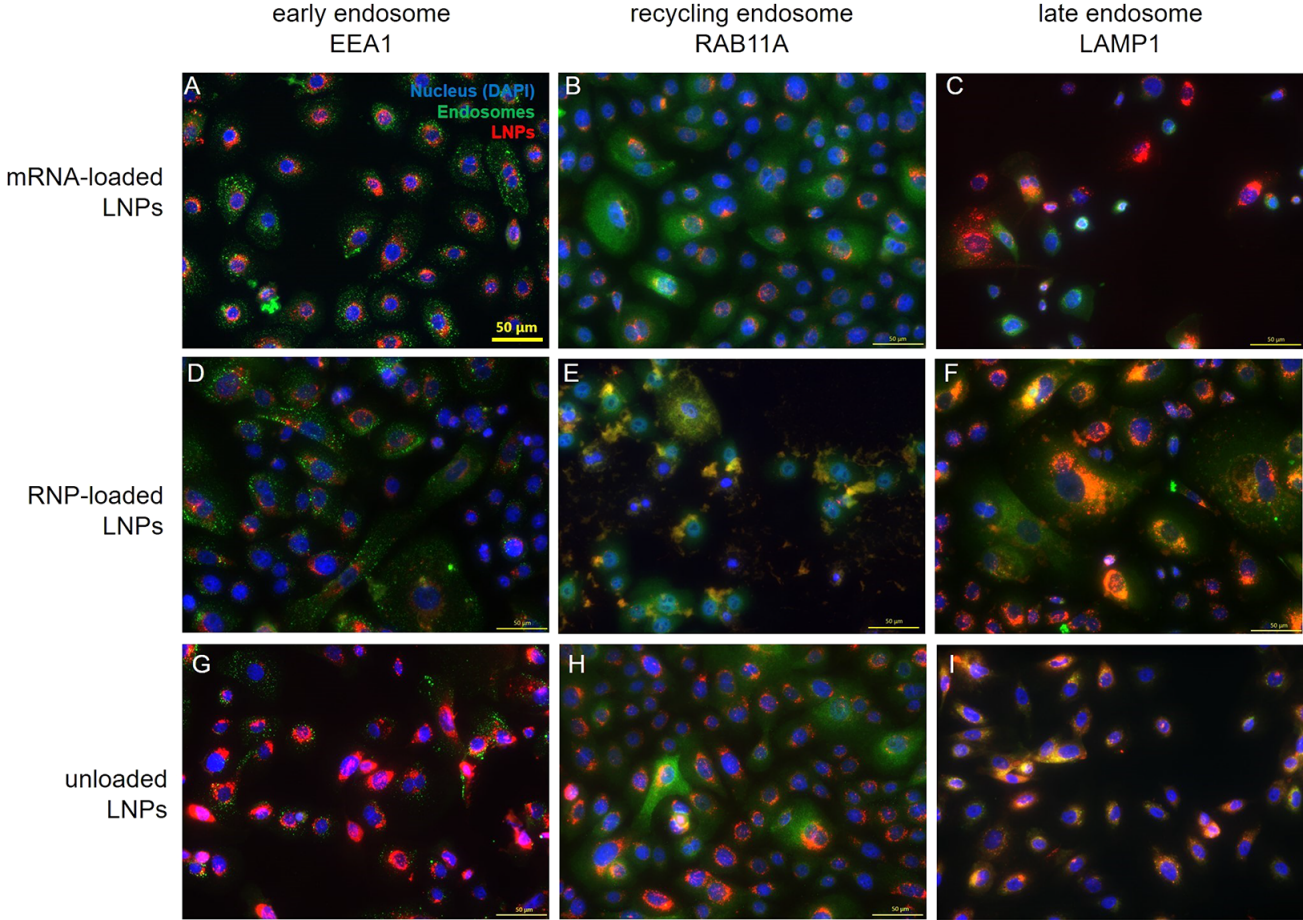
Immunofluorescence staining against EEA1 (early endosome marker), RAB11A (recycling endosome marker), and LAMP1 (late endosome marker) in primary human KCs 24 h after treatment with mRNA-loaded, RNP-loaded, and unloaded LNP. Scale bar = 50 μm.

Once internalized, LNPs enter endosomes, from which they need to escape to yield functional effects. In general, less than ≤2% eventually reach the cytosol ultimately limiting LNP efficacy and requiring higher doses to induce a therapeutic effect.^48,49^ The endosomal route of a particle once more depends on the LNP composition, size, zeta potential, and the cell type.^50,51^ Hence, we investigated the endosomal localization of mRNA and RNP-loaded LNPs in KCs (Figure 3, Figure S5) noting a different compartmentalization for these genetic cargos. RNP-LNPs predominantly colocalized in recycling (RAB11A+) and late (LAMP1+) endosomes, whereas only a few mRNA-loaded LNPs were detected in late endosomes. Interestingly, no colocalization with EEA1+ early endosomes was observed for both mRNA and RNP, which is in contrast to studies in adipocytes, fibroblasts, and HeLa.^52,53^ However, this is noteworthy as endosomal escape rates seems to be highest for early and recycling endosomes.^46,52^ To increase the potency of current and future LNP formulations, strategies related to optimized LNP composition or the application of endosomal escape enhancers^54^ are pivotal when striving for potent and well-tolerated formulations.

### Impact of the Ionizable Lipid on Gene Editing Efficacies in KC

Ionizable lipids (ILs) are key components of LNPs as they govern distinct RNA protecting effects, facilitate the cytosolic transport, and, thus, determine LNP efficacy.^55,56^ ILs possess a p*K*_a_ which ensures that the lipid is neutral under physiological conditions^39^ and positively charged at acidic pH to enable efficient entrapment of genetic cargo. By incorporating ILs, carrier-related side effects are significantly reduced while the therapeutic index can be improved by several orders of magnitude.^57,58^

Aiming to better understand the role of the IL on gene editing efficacies in KCs, we tested a variety of mRNA-loaded LNP formulations containing proprietary ILs that cover a p*K*_a_ range between 5.4–8.1 while the rest of the LNP composition remained unchanged. Since these studies were performed with mRNA only, microfluidic mixing was employed for particle preparation and loading.

**Figure 4:**
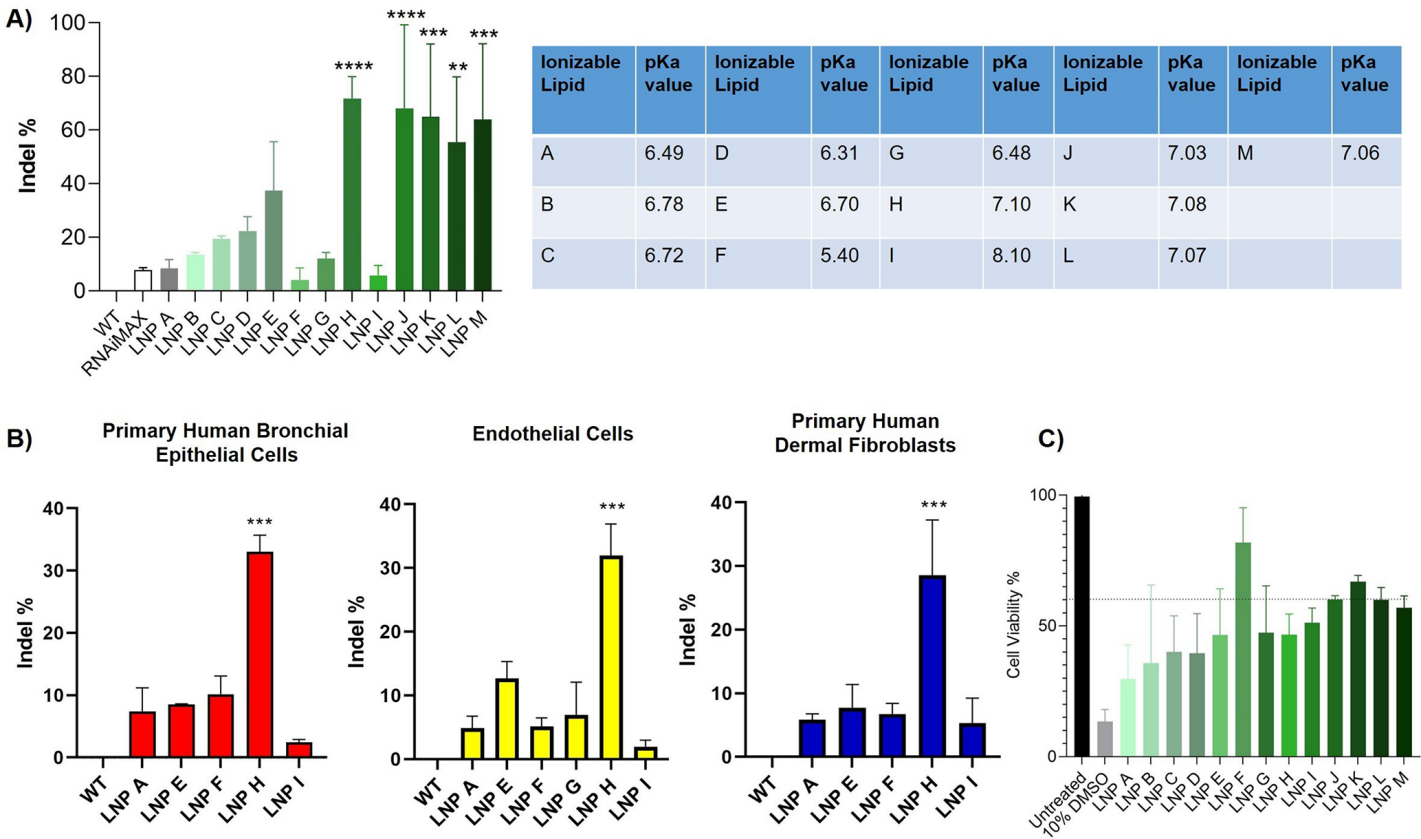
Impact of ionizable lipids and their p*K*_a_ on the frequency of indel% (normalized to wild-type (WT) cells) in (A) primary human KCs and (B) bronchial epithelial cells as well as nonepithelial cells including endothelial cells and dermal fibroblasts. (C) Cell viability of primary human KCs after a 48h treatment with the different mRNA-loaded LNP formulations. Data are presented as mean ± SD of at least three biologically independent replicates. * indicates statistical significance over RNAimax: *p < 0.05; **p < 0.01; ***p < 0.001.

While no major differences were observed for LNPs containing IL with a slightly acidic p*K*_a_, we consistently noted significantly increased editing efficacies (≥50%) for IL with p*K*_a_ ≥ 7.0 (Figure 4A). Compared to our initial MC3-based LNP formulation, gene editing efficacy increased to 72% for LNP H, 68% for LNP J, 65% for LNP K, 55% for LNP L, and 64% for LNP M, although a more pronounced donor variability was noted for the last four sets, the reason for which is unclear at this point. However, similar to the cytotoxicity data shown in Figure 2, these mRNA-loaded formulations also reduced cell viabilities by 50% after 48 h in KC monolayers (Figure 4C).

Intrigued by the fact that a more neutral p*K*_a_ increased gene editing efficacies in primary human KCs, we next investigated whether this concept is transferrable to other primary epithelial cells and cells derived from different germ layers. Indeed, we observed a similar trend in primary human bronchial epithelial cells, endothelial cells, and fibroblasts in which LNP H (p*K*_a_ 7.1) resulted in significantly higher gene editing rates compared to other LNPs (Figure 4B). Interestingly, across all tested cell types, LNPs with an IL p*K*_a_ of 8.1 yielded the lowest editing efficacies. Such a bell-shaped relationship between p*K*_a_ and potency has been previously demonstrated for siRNA, for which the p*K*_a_ optimum was ∼6.2–6.5 beyond which LNP potency declined rapidly.^43^

### In Situ Gene Editing Efficacy in Excised and Reconstructed Human Skin

Pursuing the ultimate goal of enabling *in situ* gene editing of human skin, we next tested the performance of our lead LNPs (DOPE-MC3 LNP and LNP H) in actual three-dimensional (3D) skin tissue. We used freshly excised human skin from plastic surgeries and 3D bioengineered skin models to closely recapitulate clinical scenarios. The skin models closely resemble human skin, are well-differentiated, and as such exhibit all relevant skin layers and terminal skin differentiation markers.^59^

Human skin is an important defense line of the human body and, thus, forms a very restrictive barrier which efficiently prevents the penetration of nanoparticles even in diseased states.^60^ Consequentially, when aiming for *in situ* gene editing in epidermal skin layers, a pretreatment of the skin is warranted to aid the penetration of LNPs across the stratum corneum and into the viable epidermis. This is particularly important for genetic diseases like congenital ichthyoses, which are accompanied by a thickening of the stratum corneum (hyperkeratosis).

**Figure 5:**
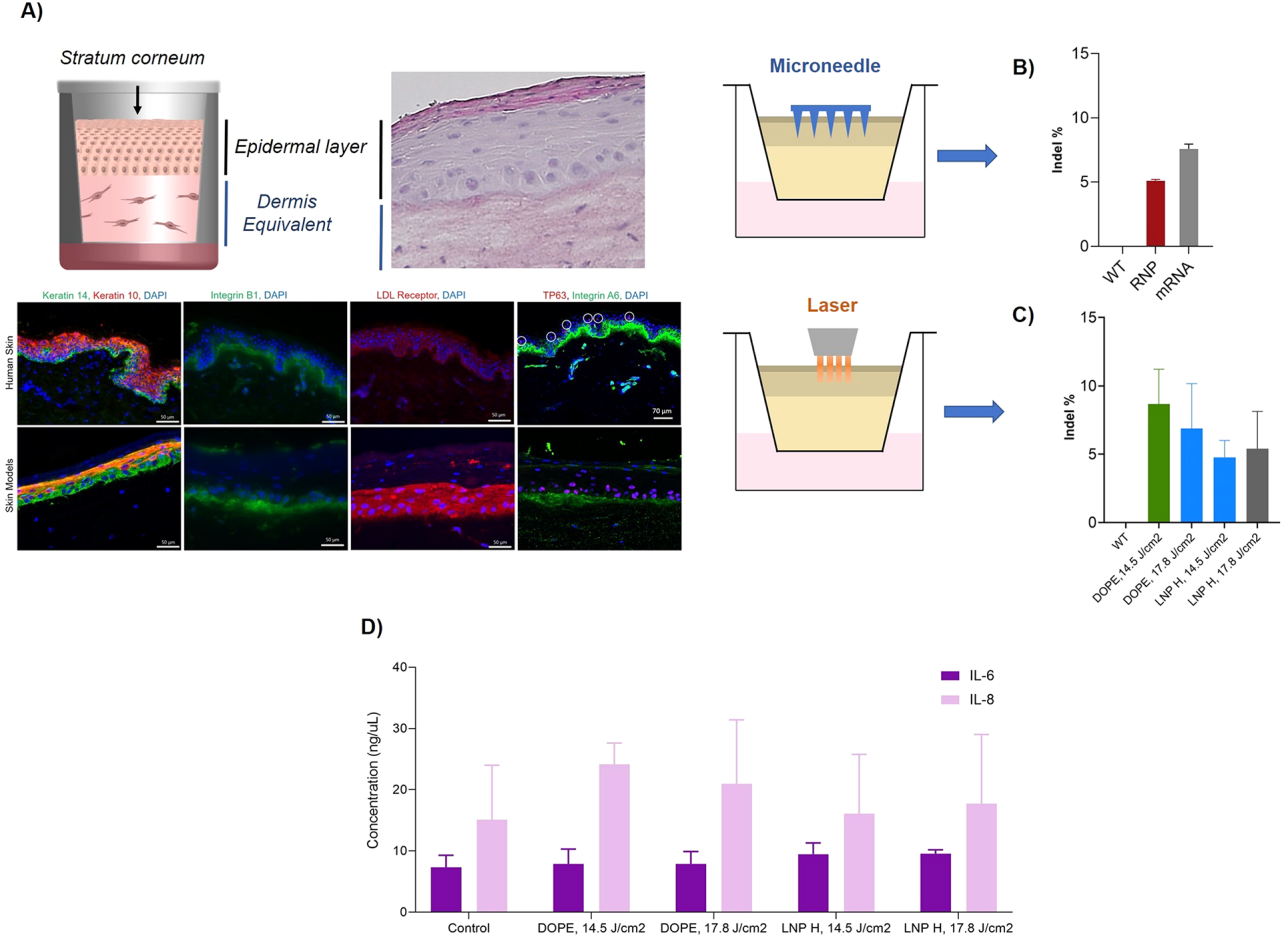
(A) Schematic representation of 3D skin models including histological and immunofluorescence staining verifying the comparability to human skin. (B) Frequency of indel% (normalized to wild-type (WT) models) of topically applied RNP- or mRNA-loaded DOPE LNP in 3D skin models after 48 h following pretreatment with 400 μm solid microneedles and (C) pretreatment with laser ablation. (D) IL-6 and IL-8 levels of untreated (control) and DOPE-LNP and LNP H treated skin models after pretreatment with laser ablation.

We utilized and compared two approaches: a microneedle-based approach (400 μm length) and a clinically approved Er:YAG fractional ablative laser (P.L.E.A.S.E. Professional) that allowed targeted pore formation in epidermal skin layers and perforation of the stratum corneum. Subsequently, RNP- or mRNA-loaded LNPs were topically applied (Figure 5A, Figures S6–S8).

Overall, we obtained *in situ* gene editing rates between 5%–12% after a single application of mRNA- or RNP-loaded LNPs in excised human skin and bioengineered 3D skin models (Figure 5A). Notably, due to limited availability of freshly excised human skin, we assessed the predictivity of 3D skin models noting matching editing rates (Figure S9). This is also particularly relevant for future proof-of-concept studies in disease models as excised skin from genodermatose patients is typically not available, rendering bioengineered disease models critical for further preclinical testing.

Overall, we did not observe significant differences between skin pretreatments with the microneedle (MN) or the laser. Varying the pulse energies of the laser also did not affect the outcomes significantly. This is in line with previous studies showing that while higher pulse energies influence the depth of the micropores, the total drug delivery does not necessarily increase.^61,62^ Both laser ablation and MN have well-documented clinical safety profiles triggering no or minor local reactions such as itching or redness.^63−67^ The skin barrier function typically regenerates within a few hours^68,69^ while pore closure occurs within 24–48 h.^70,71^ Further, phase 3 clinical trials did not show any elevated infection risks following pore induction.^69^

Surprisingly, no significant differences were observed between DOPE-LNPs (∼14% editing in KC monolayers) and LNP H (∼71% editing in KC monolayers) in actual skin tissue (Figure 5B,C). We hypothesize that this may be due to the differentiation stages KCs obtain in skin tissue which is associated with decreased LDL receptor expression^72^ resulting in less LNP uptake, hence lower editing efficacy. Another possibility is the limited distribution of the LNP in skin tissue. Also, it should be noted that primary human keratinocytes in two-dimensional (2D) exert stem-cell-like character and uniformly show high LDL receptor expression. In skin tissue, however, the cells are differentiated, forming a stratified epithelium where each epidermal layer exerts different cell activity.

It is noteworthy, however, that correcting 5%–10% of disease-causing mutations suffices to alleviate the most severe symptoms^73,74^ and restore the skin barrier function.^75^ While our current editing rates fall within this window, future studies will determine if a repeated application may further increase the *in situ* editing rates.

To assess a potential skin irritating effect of the treatments, we quantified the release of classic pro-inflammatory cytokines IL-6 and IL-8 from both skin models (Figure 5D) and excised human skin (Figure S10). In both cases, cytokine release was in the low nanogram range and no significant increase was observed for all tested conditions compared to untreated skin samples (Figure 5D).

### Cas9 RNP Shows an Extended Presence in Epithelial Cells, and Cas9 mRNA Induces No Detectable sgRNA-Dependent Off-Target Edits

When aiming for *in situ* gene editing, the safety and accuracy of the gene editing approach is imperative. Past data suggests that Cas9 RNP may induce fewer off-target edits due to rapid intracellular degradation.^76−79^ For example, Kim and co-workers demonstrated that very little RNP was still detectable after 24 h while no RNP was present after 48 h in K562 leukemia cells. In contrast, alternative Cas9 expression systems such as plasmids or mRNA cause sustained overexpression, potentially resulting in more off-target effects.

**Figure 6:**
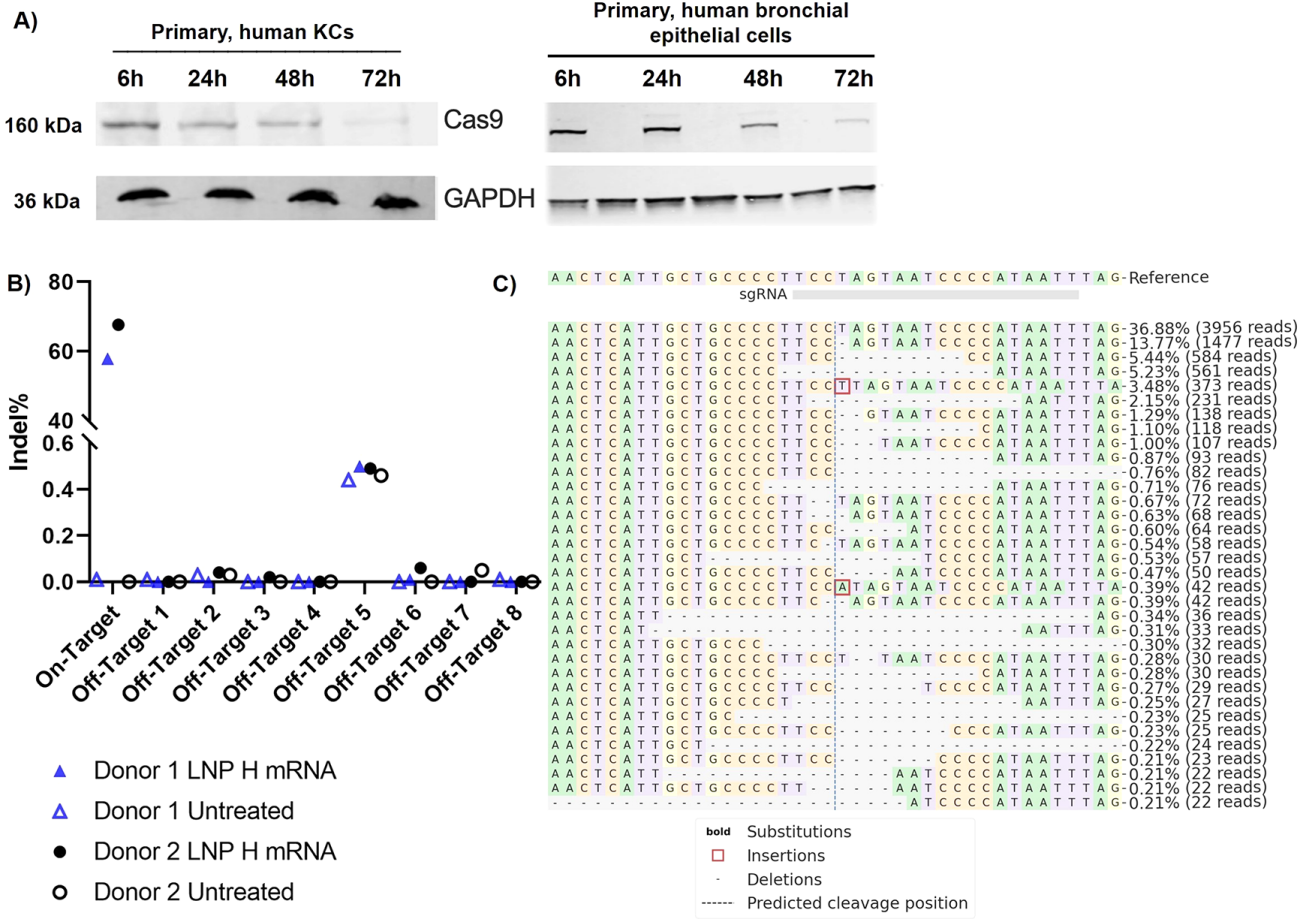
(A) Western blot analysis of Cas9 protein expression in primary human KCs and primary human bronchial epithelial cells 24, 48, and 72 h after treatment with Cas9 RNP loaded onto DOPE-LNPs. GAPDH served as housekeeper. (B) Using rhAMPseq, effective on-target editing of our model target HPRT with LNP H was confirmed while no off-target effects were observed in any of the eight predicted sgRNA-dependent off-target sites. (C) Visualization of the distribution of the most frequently identified alleles around the cleavage site for sgRNA AATTATGGGGATTACTAGGA in donor 1. Nucleotides are indicated by unique colors (A = green; C = red; G = yellow; T = purple). Substitutions are shown in bold font. Red rectangles highlight inserted sequences. Horizontal dashed lines indicate deleted sequences. The vertical dashed line indicates the predicted cleavage site.

Interestingly, we were still able to detect RNP after 72 h in primary human KCs and bronchial epithelial cells (Figure 6A) indicative of the cell-specific kinetics for RNP degradation. Previous studies mostly determined intracellular Cas9 protein presence in rapidly proliferating cancer cell lines, which may be one reason for the discrepancy. Primary epithelial cells usually proliferate at much slower rates, which results in fewer dilutive effects. The actual reason for the prolonged intracellular Cas9 protein presence in epithelial cells will require further investigations.

To gain a first indication for potential off-target edits, we assessed the frequency of off-target effects in eight predicted sgRNA-dependent off-target sites after treatment with our most efficient formulation LNP H using the rhAmpSeq analysis (Figure 6B,C). Due to similar efficiencies and the ease of manufacturing, mRNA-loaded LNPs produced via rapid mixing were selected only for all subsequent studies.

With rhAMPSeq, an allele frequency analysis was performed to identify indels and single nucleotide variants (SNVs). While the sequencing confirmed the on-target effects (see Figure 4A), no off-target edits were detected in any of the predicted off-target sites (Figures 6B, S11, & S12). We observed background indel frequencies in both treated and control samples in the very low percentile range. It should be noted that rhAMPSeq detects edits at frequencies ≥0.5% with very high specificity and sensitivity, whereas ≤0.5% is the detection limit.^80^ The indels ranging between 0.44–0.5% that occur in both treated and untreated samples are most likely due to a genomic motif that rhAMPSeq has difficulties reading, which is corroborated by the fact that this was observed across different donors.

While these data cannot fully exclude the possibility of off-target mutations in any other sites, they provide a good initial indication. Also, it should be noted that we did not use a high-fidelity Cas9. In future studies, whole genome sequencing will be required to obtain a more detailed picture. Up to now, however, it has remained unclear how many off-target effects are tolerable and if the definition of thresholds is even feasible. Nonetheless, as of today most *in vitro* and *in vivo* studies indicate very high specificity of gene editing approaches and very few if any detectable off-target mutations.^5,6^

### Lipid-Based Delivery of Base Editors Facilitates Efficient Correction of Disease-Causing Mutations in Autosomal Recessive Congenital Ichthyosis (ARCI) Patient Cells

**Figure 7:**
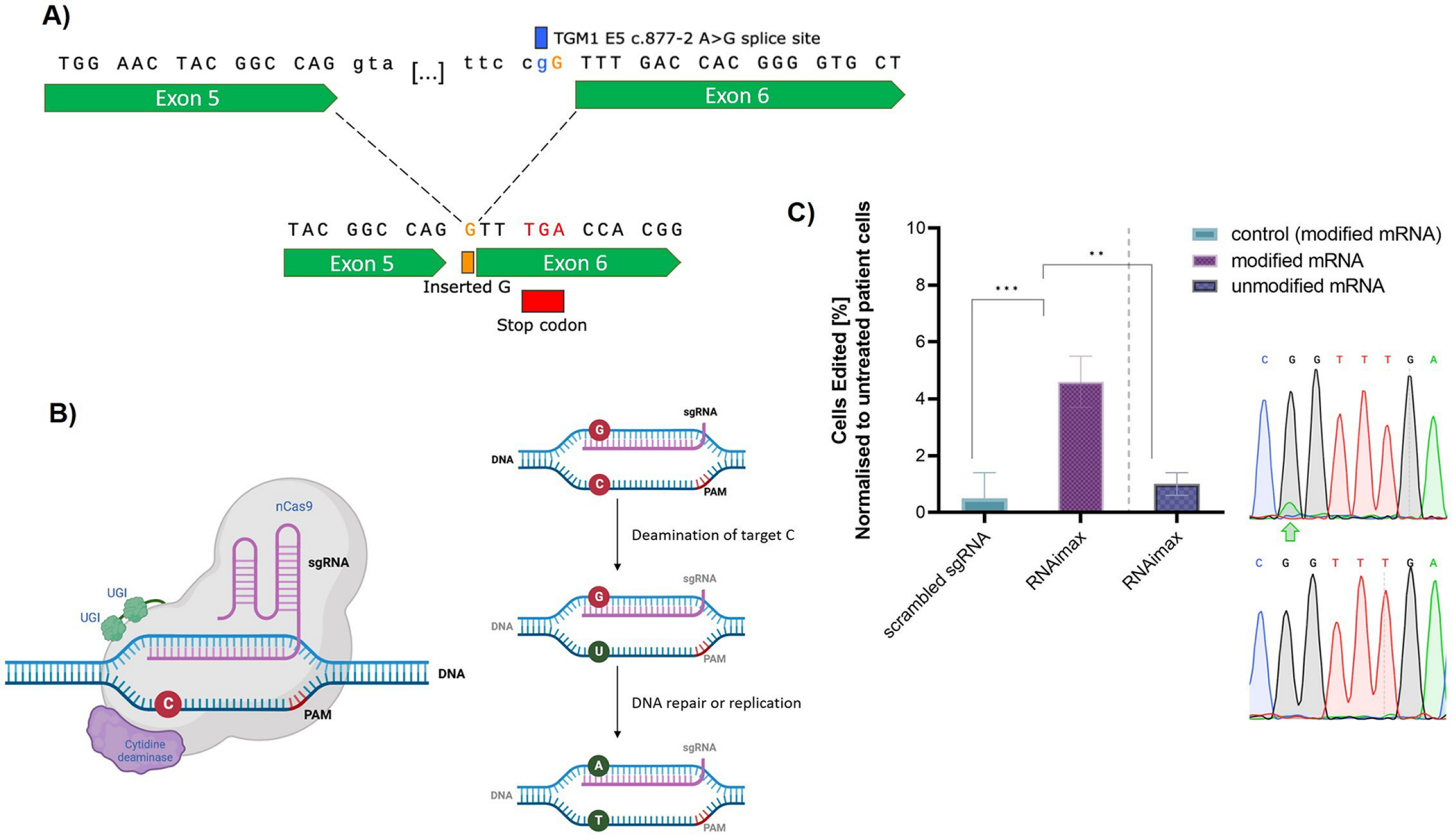
(A) The sequence of one of the most common ARCI causing mutation TGM1 c.877–2 A>G. (B) Schematic depiction of the underlying mechanism of a cytosine base editor. (C) Base editing of TGM1 c.877–2 A>G ARCI patient cells using RNAimax and NG-BE4max editor with 15 μg/mL modified and unmodified mRNA. Editing of the A>G mutation on the coding strand is displayed as the mean normalized editing rates from three technical replicates. Chromatograms of sequences after treatment with (C) RNAimax show a double peak at the target site, indicative of base editing. The edited sites are marked with a green arrow.

Ultimately, we aimed to provide a proof-of-concept that lipid-based delivery systems can correct actual disease-causing mutations. For our disease of interest, ARCI, the most common mutation is *TGM1* c.877–2 A > G, which affects up to 1/3 of ARCI patients (Figures 7A,B & S13).^81^ This splice site mutation causes a premature stop codon resulting in a truncated and thus nonfunctional version of transglutaminase 1 (TGase 1). TGase 1, however, is a critical enzyme for the cross-linking of the skin’s outermost barrier, the stratum corneum. A lack of TGase 1 results in a significantly disturbed skin barrier function characterized by increased transepidermal water loss and increased susceptibility to skin infections which can even cause life-threatening conditions especially in newborns.^82^

To correct *TGM1* c.877–2 A > G, we selected the cytosine base editor NG-BE4max, which targets the noncoding strand, thus, causing a base exchange from the mutant G back to the wild-type A on our target strand.^83,84^ We then prepared modified and unmodified base editor mRNA and assessed their editing efficacy in ARCI patient cells using RNAimax. This treatment resulted in editing rates of 4.6 ± 0.9% with the modified mRNA, while unmodified (uridine 5′-triphosphate) mRNA yielded no detectable edits (Figure 7C). The modified base we used is N1-methyl pseudouridine to replace normal uridine triphosphate during *in vitro* transcription as it has previously been suggested to enhance protein expression and reduce immunogenicity in mammalian cells and mice.^85,86^

Overall, this is an important initial proof-of-concept showing that lipid-based delivery systems can indeed correct disease-causing mutations through base editor delivery. Future studies will focus on further refining and optimizing the delivery strategy leveraging LNPs, dose titrations, and verifications of a functional restoration of gene function.

## Conclusion

In this study, we present a topically applicable *in situ* gene editing approach resulting in clinically relevant editing rates in human skin tissue via LNP-mediated gene delivery. Despite some advantages of RNP-loaded LNPs such as lower cytotoxicity, certain limitations remain including inefficient RNP encapsulation, yielding inhomogeneous LNP formulations. Also, in contrast to other reports, we detected Cas9 RNP ≤ 72 h in slow cycling epithelial cells, which overrides the argument of lower off-target edits through shorter intracellular presence. As LNPs have been initially developed for RNA delivery, further nanoparticle optimization is clearly required to unlock the potential of LNPs for RNP delivery.

While only minor LNP composition effects on gene editing rates were detected when varying helper and PEG lipids, ionizable lipids with a p*K*_a_ > 7.0 significantly increased the gene editing efficacy in primary epithelial and mesenchymal cells while no guide RNA-dependent off-target effects were detectable. Following skin pretreatment with microneedles and laser ablation to facilitate and guide intraepidermal LNP, we obtained *in situ* editing rates ≥5% in skin tissue without inducing pro-inflammatory cytokine release. Notably, it is hypothesized that correcting 5–10% of disease-causing mutations suffices to offset the most severe symptoms. Hence, the gene editing rates resulting from our one-time application are putatively in the therapeutic window, which, however, requires further validation. Finally, building onto the positive results with the cytosine base editors in ARCI patient cells, future studies will require the establishing of dose–response curves, validation of efficient and functional gene activity restoration in 3D skin disease models, and further investigation of the biocompatibility of our approach.

Taken together, this study describes a topical LNP-based approach yielding clinically relevant *in situ* gene editing of human skin that can be employed to correct disease-causing mutations to effectively treat and maybe even cure rare monogenic skin diseases.

## Experimental Details

### Materials and Primary Cells

Single guide RNAs (sgRNAs), GFP-tagged siRNA, Cas9 protein nuclease, PCR primers and probes, PrimeTime qPCR mix, rhAMP sequencing library kit, and index primers were obtained from IDT (San Jose, CA, USA). The Cas9 mRNA was purchased from TriLink Biotechnologies (San Diego, CA, USA), and the GFP-labeled Cas9 protein nuclease was purchased from GenScript (Piscataway, NJ, USA). Epilife medium and Epilife defined growth supplements, Dulbecco’s modified eagle’s medium (DMEM), fetal bovine serum, and penicillin were purchased from Fisher Scientific (Mississauga, ON, Canada). Mouse monoclonal antibody against Cas9 (ab191468) and live and dead cell assays were purchased from Abcam (Cambridge, MA, USA). ApoE4 (Apolipoprotein 4) was purchased from Peprotech (Rocky Hill, NJ, USA). Primary human keratinocytes (KCs) were isolated from excised human skin or juvenile foreskin according to standard procedures (written consent was obtained, CREB# H19-03096). KCs were maintained in Epilife media. All cells were maintained at 37 °C under a humidified atmosphere of 5% CO_2_.

### Lipid Nanoparticle (LNP) Preparation and Cas9 RNP or mRNA Loading

LNPs were prepared by injecting the lipid mixture dissolved in ethanol at appropriate ratios to a final concentration of 10 mM lipid with an aqueous phase containing Cas9 or NG-BE4max mRNA and HPRT or TGM1 sgRNA through a T-junction at a 3:1 volume ratio and an amine-to-phosphate (N/P) ratio of 6.^87^ Flow rates were set to 5 mL/min for the lipid-phase syringe and 15 mL/min for the aqueous-phase syringe containing the RNA dissolved in 25 mM sodium acetate (pH 4) culminating in an output flow rate of 20 mL/min. The resulting formulation was then dialyzed in Spectra/Por 2 12–14kD molecular weight cutoff (MWCO) dialysis tubing (Spectrum Laboratories) against 1000-fold volume of phosphate-buffered saline (pH 7.4) overnight at room temperature to remove the ethanol. Formulations were then sterile-filtered and concentrated to target nucleic acid concentrations in 10 kDa Amicon filters (Sigma-Aldrich).

For the benchtop mixing approach, empty LNPs were prepared as described above followed by cargo loading.^39^ Here, RNP complexes were formed by combining sgRNA with the Cas9 protein at 1:1 equimolar ratio in 10 mM Tris, 0.1 mM ethylenediaminetetraacetic acid (EDTA) buffer at pH 7.5 to a final working concentration of 25 μM, followed by incubation at room temperature for 5 min. For LNP-RNP complexation, 100 nM RNP (pH 7.5) was mixed with LNPs at L/R 250, 500, and 750 (LNPs amounts with initial stock of 3 mM; 0.026 μmol (L/R 250), 0.053 μmol (L/R 500), and 0.079 μmol (L/R 750)). For mRNA encapsulation, sgRNA and Cas9 mRNA were mixed with a 1:1 equimolar ratio to a 10 μg/mL final working concentration. To prepare LNP-mRNA formulations, 1 μg of 10 μg/mL working concentration was mixed with LNPs at N/P ratios of 3, 6, or 9. After initial complexation of mRNA or RNP with LNPs in sodium acetate buffer (pH 4), media were added, and ApoE was spiked in yielding a final concentration of 1 μg/mL.

The final lipid concentration was measured using a Total Cholesterol Assay kit (Wako Chemicals, Richmond, VA, USA). A typical LNP formulation would contain the following lipids: ionizable cationic lipid, phospholipid, cholesterol, and PEG-lipid at 50/10/38.5/1.5 mol %, respectively. For systems containing fluorescent labels, DiI-C18 (Invitrogen, Carlsbad, CA) was included at 0.2 mol % at the expense of cholesterol. The cationic lipid DOTAP was included at the expense of MC3 resulting in ∼71 nm (PDI 0.14) and ∼132 nm (PDI 0.16) for 20% and 40% DOTAP, respectively. Similarly, for higher PEG-lipid contents, cholesterol was decreased. Except for MC3, all other ionizable lipids were proprietary cationic lipids synthesized by NanoVation Therapeutics.

To assess the impact of buffer pH on RNP activity, we prepared the RNP solution in a buffer of varying pH (pH 4–pH 7) before adding it to the lipids that were dispersed in a pH 4 acetate buffer. Following the addition of RNP to the lipids, a neutral buffer or cell culture medium was quickly added to adjust the formulation to pH 7.4.

### LNP Characterization

#### Dynamic Light Scattering (DLS)

To determine the size and ζ potential of LNP formulations, the samples were diluted in 1 mL of sodium acetate buffer (pH 4.0) or 1 mL of Epilife media (pH 7.0), respectively. Subsequently, the number mean (d.nm), polydispersity index (PDI), and ζ potential were determined using a Zetasizer Nano ZS (Malvern Panalytical, St. Lauren, Canada).

#### Ribogreen Fluorescence Assay

To quantify the RNP and mRNA encapsulation efficiency (EE%), the Quant-iT Ribogreen fluorescence assay was performed according to the manufacturer’s instructions. Briefly, loaded LNPs were diluted in sodium acetate buffer (pH 4.0) containing Ribogreen in the presence or absence of 0.5% (w/v) Triton X-100 in Tris-EDTA buffer. Fluorescence was subsequently measured at λex = 500 nm and λem = 525 nm. Total RNP and mRNA content was then calculated from a standard curve, and EE% was calculated by comparing RNP and mRNA concentrations in the presence or absence of Triton X-100.

### Cellular Internalization Studies

#### Cellomics ArrayScan

Keratinocyte internalization was semiquantified using the Cellomics ArrayScan (ThermoFisher, Burnaby, BC, Canada). After cell seeding, 1 μg/mL siGFP-RNA was loaded onto the fluorescently labeled LNPs, being composed of DOPC, DSPC, DOPE, DSPG, and ES as ionizable lipids, with 0.5%, 1.5%, and 5% poly(ethylene glycol) (PEG) content at N/P 6. Subsequently, 3 μL of sodium acetate buffer (pH 4.0) and cell culture media were added to a final volume of 100 μL. Lastly, ApoE4 was spiked in at 1 μg/mL, followed by 10 min of incubation at room temperature. The uptake efficiency was then tested in the presence or absence of ApoE. After 24 h of treatment, the cells were washed twice with PBS and subsequently fixed with 4% formaldehyde. Fluorescence intensity was then quantified using a Cellomics ArrayScan Infinity HCS Reader.

#### Confocal Microscopy

To study the cellular uptake, 5 × 10^5^ KCs were seeded onto 35 mm glass plates overnight and were subsequently incubated with RNP complexes (GFP-Cas9 nuclease protein + sgRNA) loaded onto Dil-labeled DOPE-LNPs (L/R 500). DAPI Fluoromount-G (Southern Biotech, AL, USA) was used for nucleus staining. Live cell images were taken every 30 min for up to 3 h by using a Carl Zeiss confocal laser scanning microscope (LSM510 META NLO, Carl Zeiss Jena GmbH, Germany).

#### LNP Uptake Mechanisms

To analyze the endocytic mechanisms of LNP uptake in KCs, DiI-labeled-LNPs were added to KCs that were pretreated with 10 μM Dyngo (inhibitor dynamin-dependent cell uptake; Abcam, CatNr. ab120689), 5 μM PitStop (inhibitor clathrin-mediated cell uptake; Abcam, CatNr. ab120687), and 2 μM CytochalasinD (macropinocytosis inhibitor; Sigma-Aldrich, CatNr. C8273) and coincubated for 6 h. Subsequently, the cells were fixed with 4% formaldehyde solution, washed, and mounted with Fluoroshield containing 4′,6-diamidino-2-phenylindole (DAPI) (Sigma-Aldrich, CatNr. F6057) overnight at 4 °C. Fluorescence imaging was conducted with a Keyence BZ-X810 All-in-One Fluorescence Microscope.

#### Intracellular Trafficking

To determine the localization of LNP in endosomes, KCs were treated with empty, mRNA, and RNP DiI-labeled LNPs for 6 and 24 h. Subsequently, immunofluorescence staining was conducted as per standard protocols. Briefly, cells were fixed with a 4% formaldehyde solution washed with PBS and permeabilized with 0.5% (v/v) Triton-X-100 (VWR, CatNr. 97063-864). Blocking was achieved with normal goat serum (ThermoFisher Scientific, CatNr. PCN5000, 1:20 diluted in PBS) for 30 min at RT. Primary antibodies against EEA1 (Invitrogen, CatNr. 14-9114-82, dilution 1:1000), RAB11A (Invitrogen, CatNr. 71–5300, dilution 1:350), and LAMP1 (Abcam, CatNr. ab25630, dilution 1:1000) were added to the cells overnight at 4 °C. Then, KCs were washed and incubated with the corresponding Alexa Fluor 488-conjugated secondary antibodies (Abcam CatNr. ab150113 and ab150077, dilution 1:400). After air drying, KCs were mounted with Fluoroshield with DAPI overnight at 4 °C.

### Cell Viability Assays

#### MTT Assay

The cytotoxicity of unloaded and loaded LNPs was investigated by a 3-(4,5-dimethylthiazol-2-yl)-2,5-diphenyl tetrazolium bromide (MTT) assay. Here, 1 × 10^4^ cells KCs and HKPs were seeded in 96-well culture plates, respectively, and cultivated until ∼70% confluency. Subsequently, the cells were treated with 1 μg/mL mRNA, 100 nmol RNP-loaded, or unloaded LNPs at 37 °C for 24 h. Subsequently, 10 μL of a 5 mg/mL MTT solution was added to each well, and the plates were incubated for 4 h. The MTT formazan crystals were then dissolved in 50 μL of dimethyl sulfoxide (DMSO). Finally, the absorbance was measured in a microplate reader (BioTekuQuant, Winooski, VT, USA).

#### Live and Dead Cell Assay

To visualize the cytotoxic effect of RNP- and mRNA-loaded LNPs on the viability of KCs, a live dead cell assay (ab115347, Abcam, UK) was performed according to the manufacturer’s instructions. KCs were cultured as described and seeded in the chamber slides (177445, Thermo Fisher Scientific, USA) and treated with RNP- and mRNA-loaded LNPs (DOPE, L/R 500) for 24 and 48h. Cells were rinsed with PBS and stained with a 10X dye solution diluted in PBS and imaged using a fluorescence microscope (EVOS M5000 cell Imaging System, Thermo Fisher Scientific).

#### Cell Uptake

The cellular uptake of RNP complexes (GFP-Cas9+sgRNA) loaded onto Dil-labeled LNPs was visualized by confocal microscopy. KCs were seeded on sterile coverslips overnight and treated with RNP-loaded LNPs (DOPE, L/R 500). After 30 min, 1, 2, 8, and 24 h, cells were washed and fixed with 4% formaldehyde for 5 min at RT. Cell nuclei were stained with DAPI (ab104139, Abcam, UK), and the slides were imaged using a confocal microscope (LSM700, ZEISS).

### Gene Editing and Off-Target Analysis

#### Quantification of Genome Editing Using PrimeTime qPCR

First, 5 × 10^5^ KCs were seeded in 6-well plates. Twenty-four hours later, the cells were transfected according to the above-mentioned protocol with both RNP- and mRNA-loaded LNPs. After 48 h, the genomic DNA was isolated using the Qiagen DNeasy Blood&Tissue Kit (Toronto, ON, Canada) according to the manufacturer’s protocol. Then, the genomic DNA was amplified by PCR using 10 μM HPRT primers and 5 μM of reference and drop-off probes, PrimeTime master mix, and water at a final reaction volume of 20 μL. The drop-off probe is designed to bind the wild-type template only and target the predicted cut sites. A PCR program was run with an initial denaturation of 95 °C for 3 min, followed by 40 cycles of 95 °C for 15s, and annealing/extension at 60 °C for 1 min. Gene editing efficiency (= indel formation) of the formulations was determined by ΔΔCT values of drop-off and reference probes and normalized to the wild-type cells.

#### In Situ Gene Editing Efficacy

To evaluate the gene editing efficacy *in situ* freshly excised and reconstructed human skin was utilized. 3D bioengineered skin models were generated according to previously published procedures.^59^ First, primary human fibroblasts and keratinocytes were isolated from juvenile foreskin (CREB approval #H19-03096). Then, 3 × 10^5^ fibroblasts were embedded in a matrix consisting of fetal bovine serum and bovine collagen I (PureCol, Advanced BioMatrix, San Diego, USA) at a neutral pH. After solidification, primary human keratinocytes (4.2 × 10^6^ per model) were seeded on top. After 24 h, the skin models were lifted to the air–liquid interface and further cultivated with media changes every other day. At day 14, the models were treated as described below. Alternatively, excised human belly skin was obtained from plastic surgeries (CREB approval #H19-03096). The excised skin was cleaned from adipose layers and punched into 2 cm discs. To facilitate intraepidermal delivery, the skin was then pretreated with 400 μm long solid microneedle arrays and subsequently mounted onto Franz diffusion cells (static-type, volume 12 mL, diameter 15 mm).^88^ The stratum corneum was facing the air and the dermis in contact with PBS pH 7.4, which served as receptor fluid. After 30 min of equilibration time, 50 μL of DOPE-LNPs (5 mM) loaded with Cas9 mRNA or RNP complexed with *HPRT* sgRNA was topically applied. After 48 h, the skin sections were removed from the Franz cell system followed by overnight incubation at 4 °C in Dispase (Worthington Biochemical Corp, Lakewood, NJ, USA) for dermis-epidermis separation. On the next day, gDNA was isolated from the epidermal layer, as described above.

For laser-assisted microporation, skin models were pretreated using the P.L.E.A.S.E device (Precise Laser Epidermal System, Pantec Biosolutions AG, Liechtenstein) with the following parameters: with 2 pulses per pore, pore density of 5%, array size of 14 mm, pulse duration of 125 us, pulse energy or fluence of 14.5 J/cm^2^ (repetition rate of 300 Hz, 1.1 W), and 17.8 J/cm^2^ (repetition rate of 200 Hz, 0.9 W). Subsequently, 50 ul of DOPE and LNP H formulations loaded with Cas9 mRNA were topically applied and incubated for 48 h, followed by gDNA isolation.

To visualize the micropores in the skin, excised human skin was topically treated with Dil-labeled LNPs for 24h. The nonlaser-treated skin samples served as control. The tissue blocks were then cryosectioned using a cryostat (Leica CM 1520) and imaged using a fluorescence microscope.

#### ELISA

Media from skin models and excised human skin after microneedle or laser and/or LNP treatment were diluted at a ratio of 1:100 up to 1:500 and probed for cytokine expression using the IL6 or IL8 Human Uncoated ELISA Kit (ThermoFisher, Burnaby, BC, Canada) as directed by the manufacturer. Absorbance was measured at 450 nm in a microplate reader (BioTekuQuant, Winooski, VT, USA).

#### Western Blot

One × 10^5^ KCs were seeded in 12-well plates and transfected with RNP (L/R 500)-loaded DOPE-LNP for 24, 48, and 72h. Subsequently, the cell lysates were extracted with radioimmunoprecipitation assay buffer containing proteinase inhibitor cocktail, incubated for 30 min on ice, and centrifuged at 14,000 rpm for 30 min. The amount of proteins was quantified using Bradford assay (Bio-Rad, Mississauga, ON, Canada). Ten micrograms of protein were then separated using an 8% gradient polyacrylamide gel under reducing conditions. Protein was then transferred to the nitrocellulose membrane and after washing the blots were incubated with a rabbit polyclonal antibody against Cas9 (1:1000 in TBST) overnight. To normalize detected protein levels, equal loading across the gel was confirmed by probing for GAPDH. The membrane was incubated with rabbit monoclonal antibody anti-GAPDH (1:5,000 in TBST) overnight, and detection was done with fluorescence dye conjugated goat antirabbit secondary antibody (1:5,000 in TBST, 1 h incubation) on the Odyssey CLx imaging system (LI-COR).

#### Off-Target Effect Analysis and rhAMP Sequencing

Potential off-target binding sites of the gRNA were nominated using GUIDEseq as described previously by Tsai et al.^89^ Here, we focused on the eight most likely off-target sites (Table S1) based on which the rhAMP Seq amplification and index primer panels were selected and prepared according to the manufacturer’s protocol (IDT, Coralville, IA, USA). KCs were treated with mRNA-loaded LNP H for 48 h as described above. Subsequently, the genomic DNA was amplified by PCR using 50 μM rhAMP reverse and forward primer pool, 5 μL of 4X library mix, and water for the final reaction volume of 20 μL. The first PCR used 14 cycles with an annealing temperature at 61 °C for each sample. The PCR products were immediately cleaned with Agencourt AMPure XP beads (Beckman Coulter, Canada) followed by washing with 80% ethanol and drying at room temperature for 3–5 min. Next, for indexing PCR, 5 μM samples of both i5 and i7 indexing primers were used along with 5 μL of library mix and water for the final reaction volume of 20 μL. Amplification was performed using 24 cycles with an annealing temperature of 60 °C. The indexed PCR products were pooled and again cleaned with Agencourt AMPure XP beads as mentioned before. Finally, the indexed libraries were quantified and sequenced on an Illumina (Illumina, San Diego, CA, USA) MiSeq (2 × 150 bp) sequencer (San Diego, CA, USA). rhAMP Seq sequencing data for all the samples were analyzed using the rhAMP analysis Tool (IDT, Coralville, Iowa, USA).

#### In Vitro Base Editor mRNA Transcription

BE4max-NG plasmid (pBT376, plasmid #125617) containing T7-SV40(NLS)-Apobec-1-Cas9(N) was purchased from Addgene and was linearized with PmeI (NEB, Whitby, ON, Canada). RNAs were in vitro transcribed using T7 RNA polymerase and GTP, CTP, ATP, and N1-methyl pseudo-UTP (ChemilyBio, Peachtree Corners, GA, USA). 5′ Cap and poly(A) tail were added post-transcriptionally with ScriptCap m^7^G Capping System and A-Plus Polymerase Tailing Kit (CellScript, Madison, WI, USA). RNA was purified by using a Qiagen RNeasy Kit (Toronto, ON, Canada). The integrity of the RNA was verified by gel analysis.

#### Base Editing of ARCI Patient Cells

The ARCI patient cells harboring the disease-relevant mutation c.877–2 A > G were provided by Dr. Keith Choate (Dermatology, Yale University). 1 × 10^5^ ARCI cells were seeded and subsequently treated with a final concentration of 15 μg/mL sg/mRNA encapsulated in the lipid transfection agent RNAiMax. Scrambled sgRNA served as a negative control. After 48 h, gDNA was extracted and amplified, followed by Sanger sequencing with PCR (Table S1). PCR was performed in a 25 μL reaction volume with Q5 High-Fidelity Polymerase as 2X Master Mix. Primers TGM1-E5 were used at a final concentration of 0.5 μM with a total amount of genomic DNA template of 20 ng. PCR amplicon integrity was confirmed with agarose gel electrophoresis. Twenty microliters of the amplicon were purified using the QIAquick PCR Purification Kit. A sample of each amplicon was loaded on a 1% SYBR-safe stained agarose gel along a 1000 bp ladder to confirm the integrity and size of the DNA. Visualization was done on a Gel Doc XR+ imaging system. Edits were determined by Sanger sequencing.^90^

### Statistical Analysis

Statistical analysis was performed using Prism Graphed 9.5 software (San Diego, CA, USA). Each experiment was performed at least in triplicate, and results are shown as mean ± standard deviation (SD). The statistical significance was determined using one-way analysis of variance (ANOVA) followed by Dunnett or Tukey’s multiple comparison test. *p*-Values ≤ 0.05 were considered statistically significant. For base-editing experiments, the statistical significance was assessed with a two-way multiple comparison ANOVA.

## Supporting Information Available

**Table S1.** Primer sequences, single guide RNA sequences and probes. **Figure S1**. Physicochemical characterization of LNP. **Figure S2.** Principle of PrimeTime qPCR. **Figure S3.** Indel frequency and viability in keratinocytes after treatment with DOTAP-LNP. **Figure S4.** Live dead staining of untreated human keratinocytes. Figure S5. IF staining of endosomal markers after 6h treatment with LNP. **Figure S6.** Skin cross sections after laser ablation. **Figure S7**. Distribution of DiI-LNP in skin models after microneedle and laser pretreatment. **Figure S8**. Representative histological staining of skin models after pretreatment with solid microneedles. Figure S9. Indel frequency in skin models and human skin after LNP and RNAimax treatment. **Figure S10**. Cytokine release from human skin after pretreatment with microneedles and laser. **Figure S11**. rhAMP-Seq results after treatment of keratinocytes with Cas9 mRNA-loaded LNP H. **Figure S12**. Distribution of the most frequently identified alleles around the cleavage site for the sgRNA AATTATGGGGATTACTAGGA. **Figure S13**. Characteristics of ARCI patient cells (PDF)
