# Supplementary material for: Lipid Nanoparticle-Mediated Hit-and-Run Approaches Yield Efficient and Safe In Situ Gene Editing in Human Skin
Source: ACS Nano. 2023 Nov 2;17(21):22046–59. doi: 10.1021/acsnano.3c08644 (PMC10655174; doi:10.1021/acsnano.3c08644)
Supplement: Supplementary file 1 — nn3c08644_si_001.pdf [file nn3c08644_si_001.pdf]

Supporting Information

Lipid Nanoparticle Mediated Hit-and-Run Approaches Yield Efficient and Safe *In Situ* Gene Editing in Human Skin

Juliana Bolsoni[1↓], Danny Liu[1↓], Fatemeh Mohabatpour[1↓], Ronja Ebner[1], Gaurav Sadhnani[2], Belal Tafech[1], Jerry Leung[3], Selina Shanta[1], Kevin An[4], Tessa Morin[1], Yihang Chen[3], Alfonso Arguello[5], Keith Choate[6], Eric Jan[3], Colin J.D. Ross[1], Davide Brambilla[5], Dominik Witzigmann[4], Jayesh Kulkarni[4], Pieter R. Cullis[3], Sarah Hedtrich[1,2,7,8*]

[1] Faculty of Pharmaceutical Sciences, University of British Columbia, 2405 Wesbrook Mall, Vancouver, BC, V6T 1Z3, Canada

[2] Berlin Institute of Health @ Charité Universitätsmedizin, 10117 Berlin, Germany

[3] Department of Biochemistry and Molecular Biology, University of British Columbia, 2350 Health Sciences Mall, Vancouver, BC, V6T 1Z3, Canada

[4] NanoVation Therapeutics, 2405 Wesbrook Mall, Vancouver, BC, V6T 1Z3, Canada

[5] University of Montréal, Faculty of Pharmacy, Montréal, Quebec, H3T 1J4, Canada

[6] Departments of Dermatology, Genetics, and Pathology, Yale University School of Medicine, New Haven, CT 06510, USA

[7] Department of Infectious Diseases and Respiratory Medicine, Charité - Universitätsmedizin Berlin, corporate member of Freie Universität Berlin and Humboldt Universität, 10117 Berlin, Germany

[8] Max-Delbrück Center for Molecular Medicine in the Helmholtz Association (MDC), 13125 Berlin, Germany

[↓] These authors contributed equally to this work.

***Corresponding Author:** Dr. Sarah Hedtrich, Berlin Institute of Health @ Charité Universitätsmedizin, Berlin, Germany, Lindenberger Weg 80, 13125 Berlin, Germany. Email: sarah.hedtrich@bih-charite.de, phone: +4930450541015.

Table S1. Primer sequences, single guide RNA sequences and probes.

**HPRT single guide (sg) RNA sequence**	
mA*mA*mU*rUrArUrGrGrGrGrArUrUrArCrUrArGrGrArGrUrUrUrUrArGrArGrCrUrArGrArArAr UrArGrCrArArGrUrUrArArArArUrArArGrGrCrUrArGrUrCrCrGrUrUrArUrCrArArCrUrUrGrArArA rArArGrUrGrGrCrArCrCrGrArGrUrCrGrGrUrGrCmU*mU*mU*rU	
**HPRT primers for qPCR and T7E1 Assay:**	
HPRT Forward Primer	AAGTGCCTTGTCTGTAGTGTC
HPRT Reverse Primer	ATTTCATCCGTGCTGAGTGT
**HPRT probes for qPCR:**	
Exon Location: 8 – 9, Amplicon: 149, Ref. Seq #: NM_00019, HEX/FAM	
**rhAmpSeq Primers**	
5’-/rhSeq-r/CAT CTT CCG ATG GCC TTT ATrG GAA A/GT3/-3’	
5’-/rhSeq-r/CAT TTC ATC CGT GCT GAG TrGT ACC A/GT4/-3’	
5’-/rhSeq-r/CAA ATG GAC GTG TGT AGA GCrC AGA C/GT4/-3’	
5’-/rhSeq-r/GGC TCC CGA ATC ATC AArG TCA A/GT4/-3’	
5’-/rhSeq-r/ACT AGG TCA AGA AGC ATC AGT rCCC AA/GT2/-3’	
5’-/rhSeq-r/TAC ACA AGG AGA ACC ACA GArC TGA C/GT3/-3’	
5’-/rhSeq-r/ACA GTG ATT AAT GTC TCTC GCT TTT rCTG/GT1/-3’	
5’-/rhSeq-r/AAT CCA CAG TCA AGA TGC ArGA ACA /GT1/-3’	
5’-/rhSeq-f/CAG GTC TCA GAA CTG TCC TTrC AGG T/GT1/-3’	
5’-/rhSeq-f/TGA ACC AAT CCC TAC CAT CTrC CTT T/GT1/-3’	

**rhAmpSeq Index Primers**			
rhAmpSeq i7 Index Primers, Index 1	CTTGTCGA	rhAmpSeq i5 Index Primers, Index 1	CTCGTTCT
rhAmpSeq i7 Index Primers, Index 2	TTCCAAGG	rhAmpSeq i5 Index Primers, Index 1	CTCGTTCT
rhAmpSeq i7 Index Primers, Index 2	CGCATGAT	rhAmpSeq i5 Index Primers, Index 1	CTCGTTCT
rhAmpSeq i7 Index Primers, Index 4	ACGGAACA	rhAmpSeq i5 Index Primers, Index 1	CTCGTTCT
rhAmpSeq i7 Index Primers, Index 5	AACTGAGC	rhAmpSeq i5 Index Primers, Index 1	CTCGTTCT
rhAmpSeq i7 Index Primers, Index 6	CTTGTCGA	rhAmpSeq i5 Index Primers, Index 2	TGGAAGCA
rhAmpSeq i7 Index Primers, Index 7	TTCCAAGG	rhAmpSeq i5 Index Primers, Index 2	TGGAAGCA
rhAmpSeq i7 Index Primers, Index 8	CGCATGAT	rhAmpSeq i5 Index Primers, Index 2	TGGAAGCA
rhAmpSeq i7 Index Primers, Index 9	ACGGAACA	rhAmpSeq i5 Index Primers, Index 2	TGGAAGCA
rhAmpSeq i7 Index Primers, Index 10	AACTGAGC	rhAmpSeq i5 Index Primers, Index 2	TGGAAGCA
rhAmpSeq i7 Index Primers, Index 11	CTTGTCGA	rhAmpSeq i5 Index Primers, Index 3	AGTCGAAG

rhAmpSeq i7 Index Primers, Index 12	TTCCAAGG	rhAmpSeq i5 Index Primers, Index 3	AGTCGAAG
rhAmpSeq i7 Index Primers, Index 13	CGCATGAT	rhAmpSeq i5 Index Primers, Index 3	AGTCGAAG
rhAmpSeq i7 Index Primers, Index 14	ACGGAACA	rhAmpSeq i5 Index Primers, Index 3	AGTCGAAG
rhAmpSeq i7 Index Primers, Index 15	AACTGAGC	rhAmpSeq i5 Index Primers, Index 3	AGTCGAAG

**Base Editing TGM1 c.877-2 A>G**		
Component	***Editing Strategy***	***Sequence 5’ to 3’***
sgRNA	BE4max-NG	crRNA CAAACCGGAAGGAGGGAUGG
sgRNA	Negative Control Scrambled #1 mod	crRNA GCACUACCAGAGCUAACUCA
**Primers for PCR-amplification and Sanger sequencing**		
Name	***Sequence 5’ to 3’***	
TGM1-E5 forward	CAGGGTCACATACACCCAGAGGAG	
TGM1-E5 reverse	GAGGGAGACCCCTTCCTGAAGTATC	

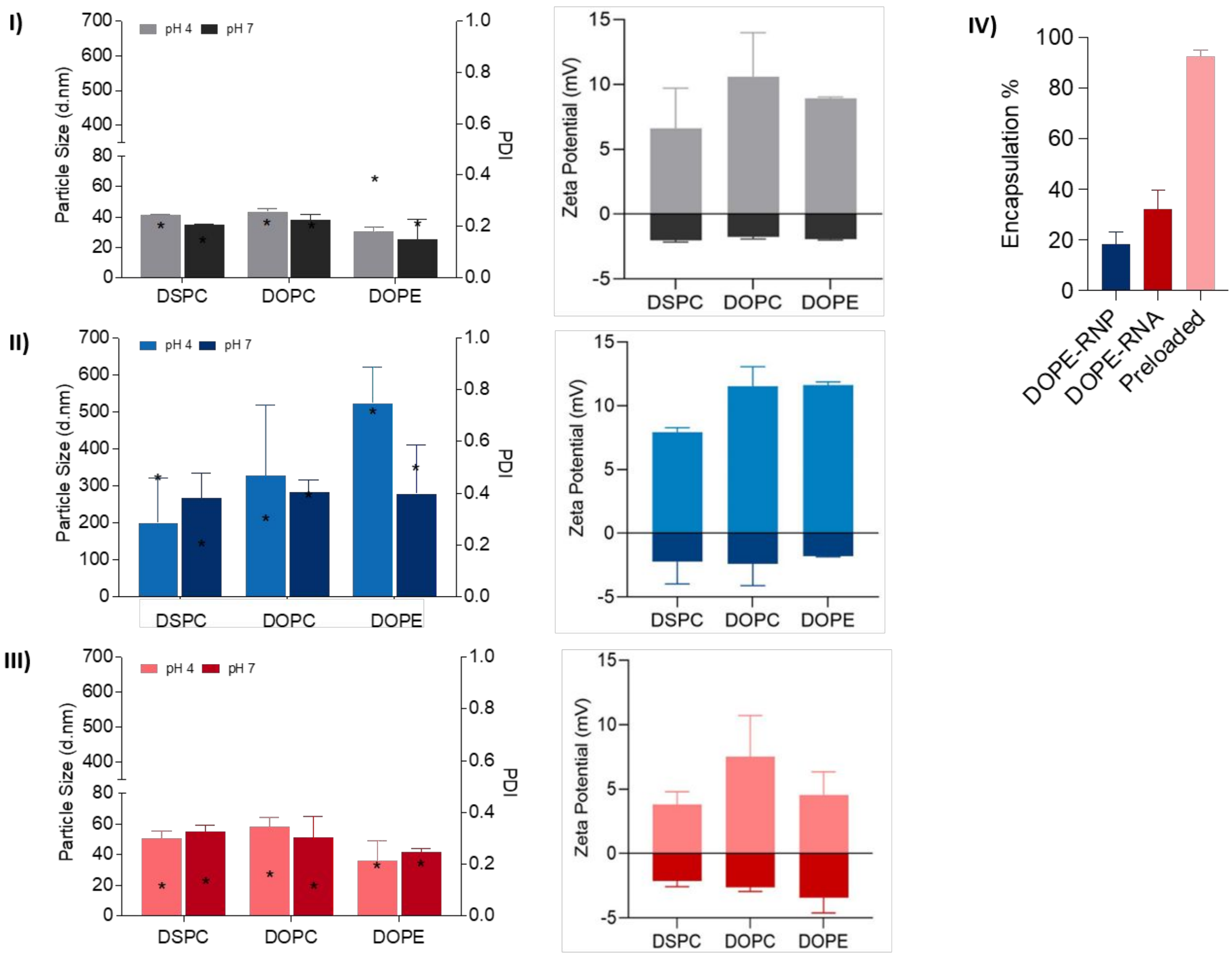

Figure S1. The hydrodynamic diameter (d.nm), polydispersity index (PDI), and Zeta Potential (mV) of ***i)*** unloaded, ***ii)*** RNP-loaded, and ***iii)*** mRNA-loaded LNPs determined by dynamic light scattering. Data are depicted as the mean value of three independent measurements ± standard deviation (SD). DOPE-LNP encapsulation efficiencies (EE%) for Cas9 RNP and mRNA following bench-top mixing and microfluidic mixing (preloaded) as determined by ***iv)*** Ribogreen® assay.

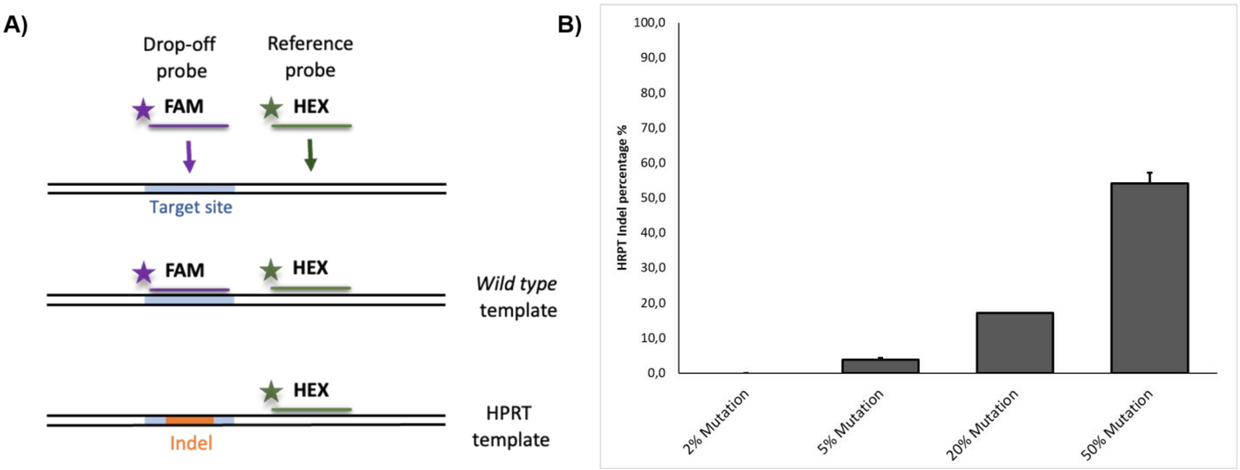

Figure S2. A) Scheme summarizing the principle of a PrimeTime qPCR assay. **B)** Validation of the PrimeTime qPCR setup to detect indel formation. Assay validation was done by the addition of pre-synthesized genomic blocks to control genomic DNA samples of unedited samples.

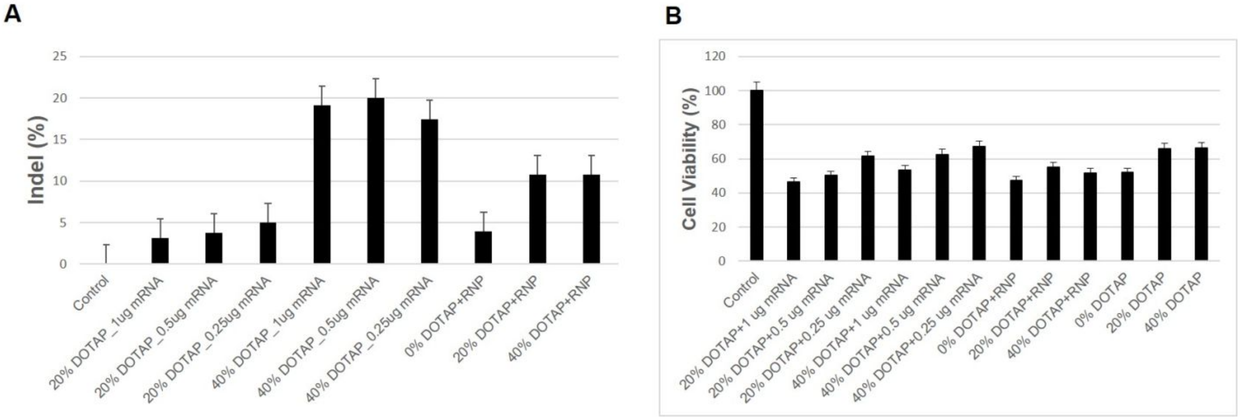

Figure S3. A) Frequency of indel % (normalized to wild-type (WT) cells) in the model gene HPRT after transfection of primary human keratinocytes (KCs) with 0%, 20%, or 40% DOTAP-containing LNP loaded with mRNA (N/P 6) or RNP (L/R 500). Data are presented as the mean of four biological replicates ± SD. **B)** Cell viability of KCs 48 h after exposure to DOTAP-containing LNP loaded with mRNA or RNP, respectively. Data are presented as the mean of three biological replicates ± SD.

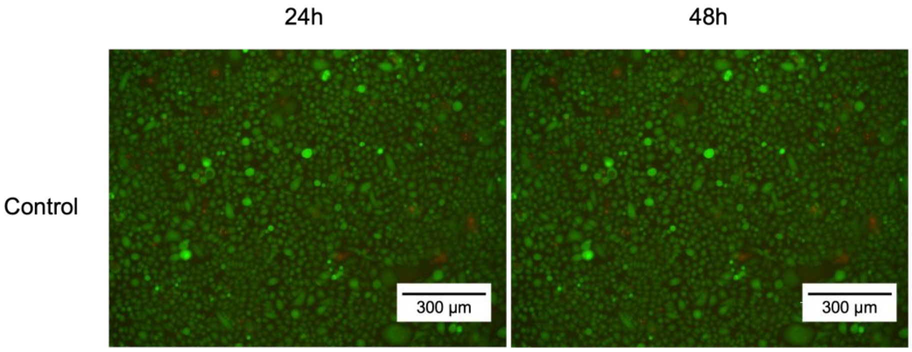

Figure S4. Live dead staining of untreated human primary keratinocytes.

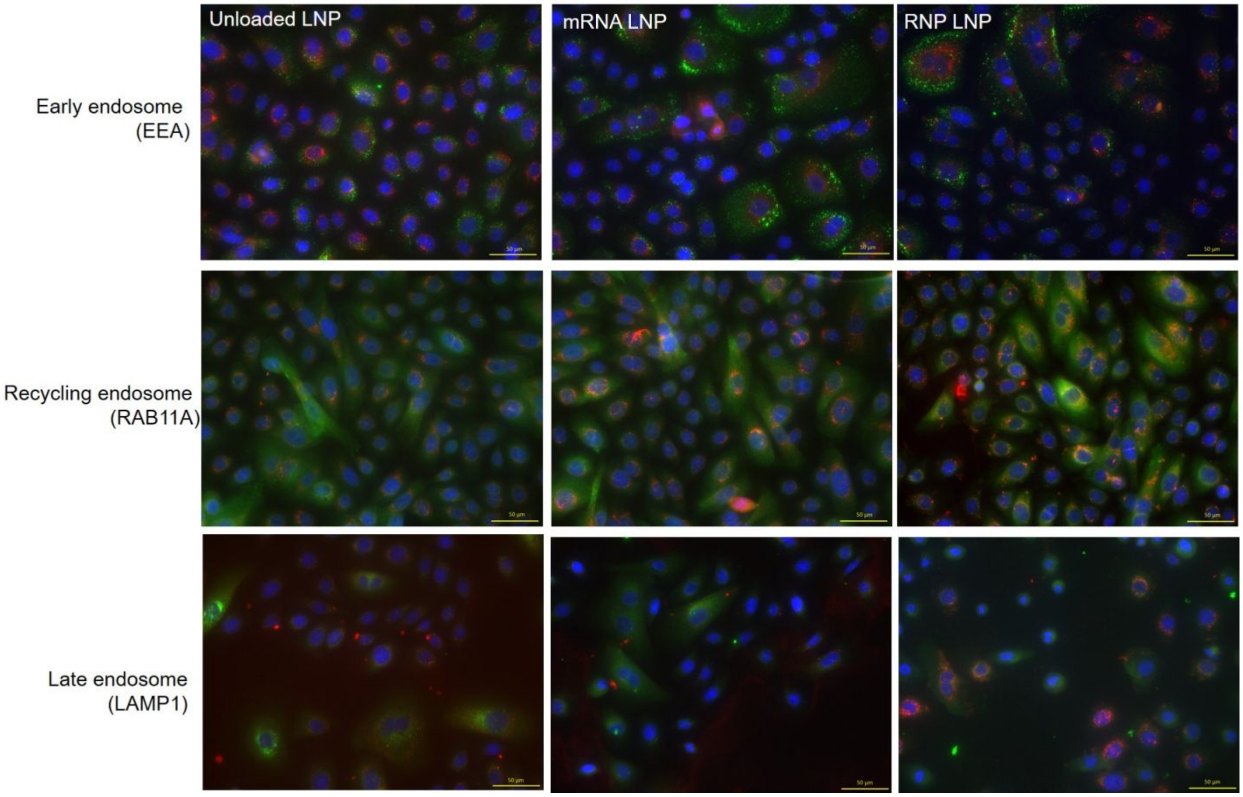

Figure S5. Immunofluorescence staining against EEA1+ (early endosome marker), RAB11A (recycling endosome marker), and LAMP1 (late endosome marker) in primary human keratinocytes 6 hours after treatment with unloaded, mRNA-loaded, and RNP-loaded LNP.

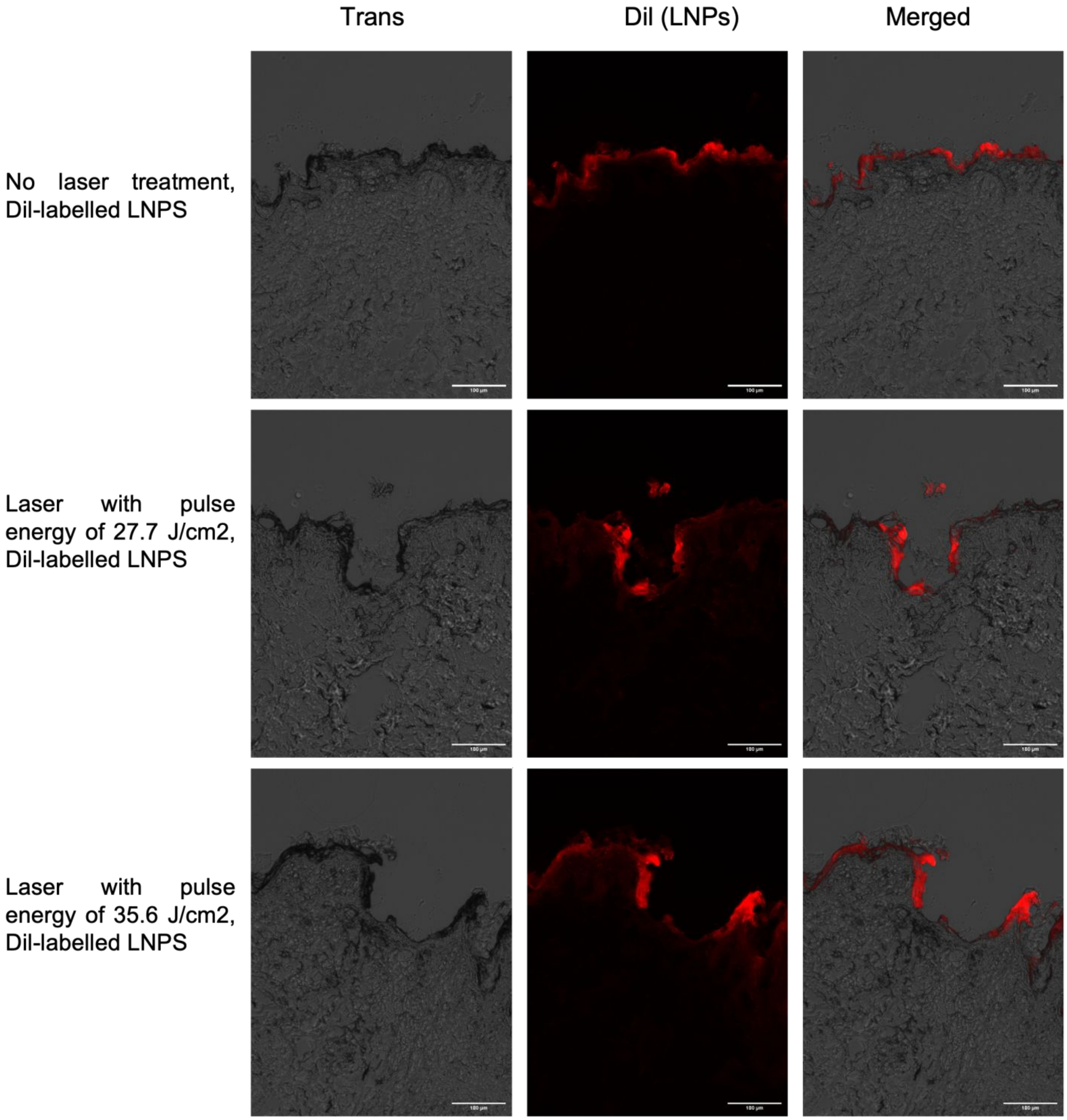

Figure S6. Skin cross-section of excised human skin after laser-induced microablation confirming the generation of the micropores.

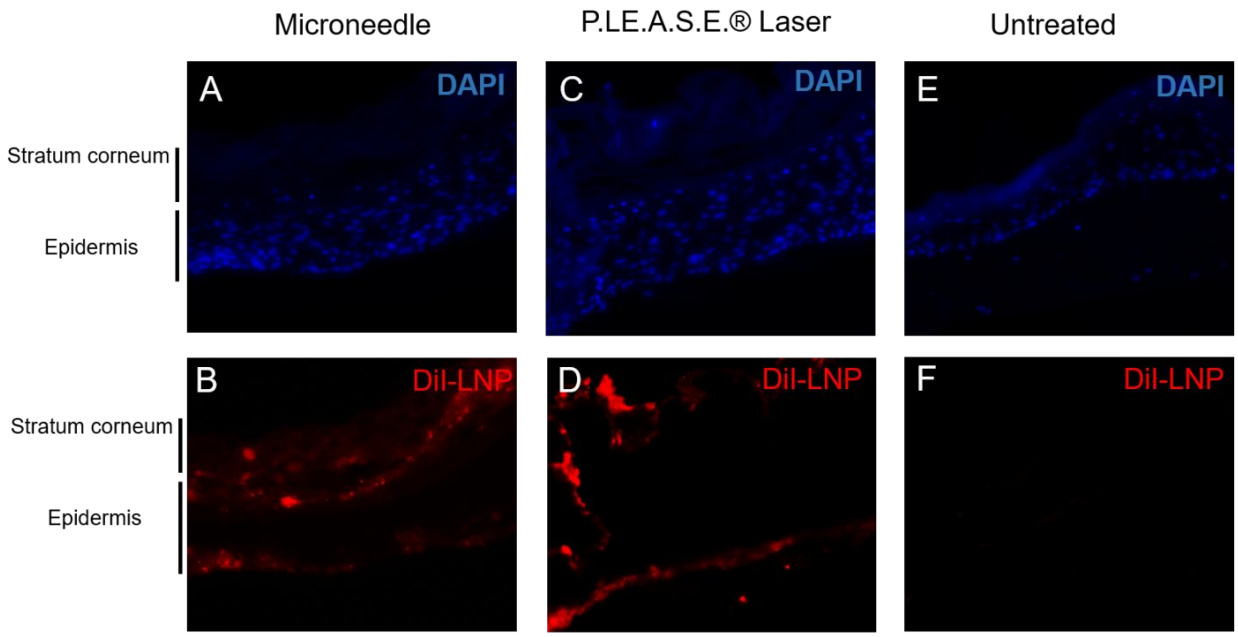

Figure S7: Distribution of DiI-LNP in 3D skin models after microneedle (A-B) and laser (C-D) treatment. DiI-LNPs were added to skin models and incubated for 24 hours, after which skin models were frozen, cryo-sectioned, and imaged with a Keyence BZ-X810. E & F show cross-sections of untreated skin models.

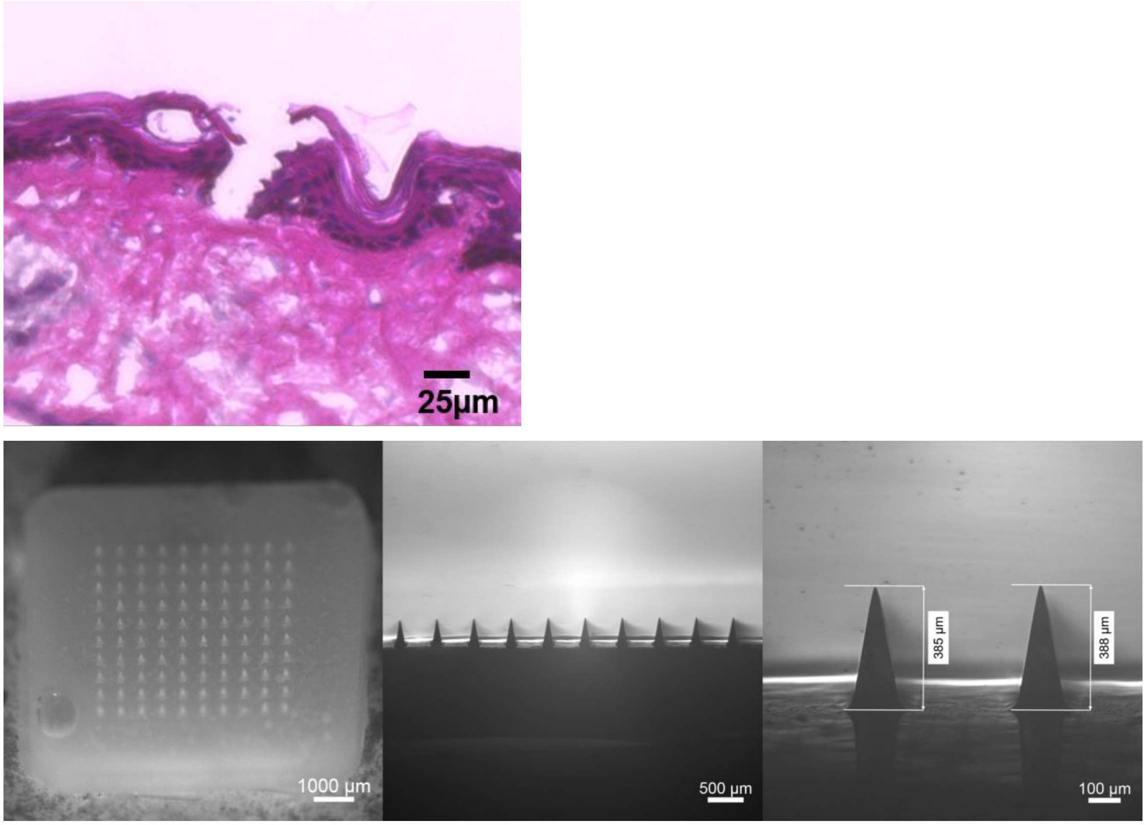

Figure S8. Top: Representative histological hematoxylin & eosin staining of bioengineered 3D skin models after pre-treatment with solid microneedles showing the opening that is restricted to the epidermis. Bottom: Representative images of the 400 µm solid microneedle array that was used for the skin pre-treatment.

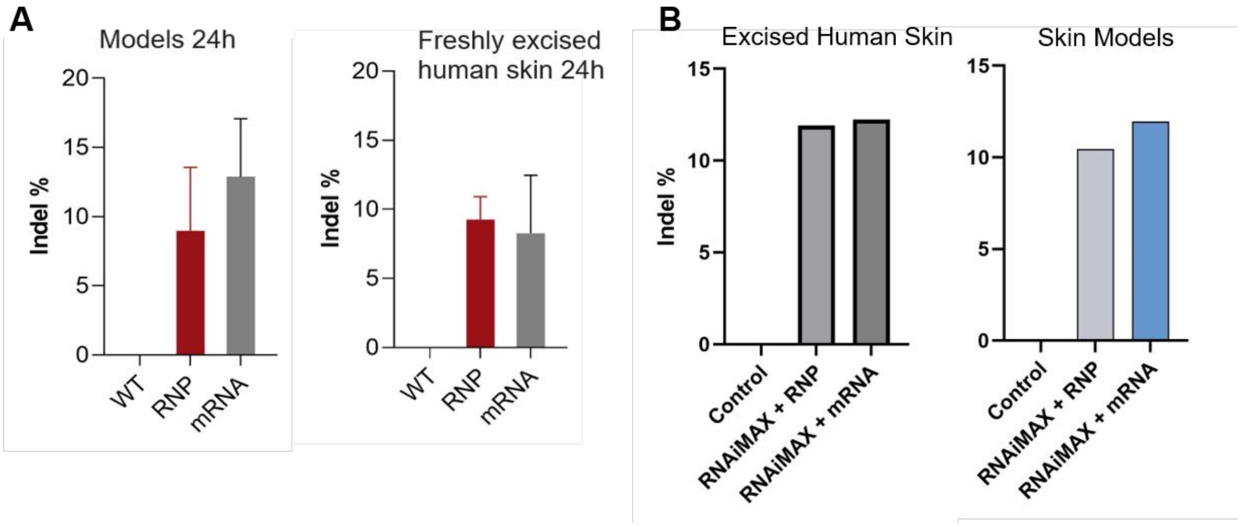

Figure S9. A) Frequency of indel % (normalized to wild-type (WT)) in bioengineered 3D skin models (left) and freshly excised human skin (right) after pre-treatment with microneedles. n=3. **B)** Frequency of indel % (normalized to wild-type (WT)) in freshly excised human skin and bioengineered 3D skin models after transfection with Cas9 mRNA or RNP encapsulated in RNAiMax.

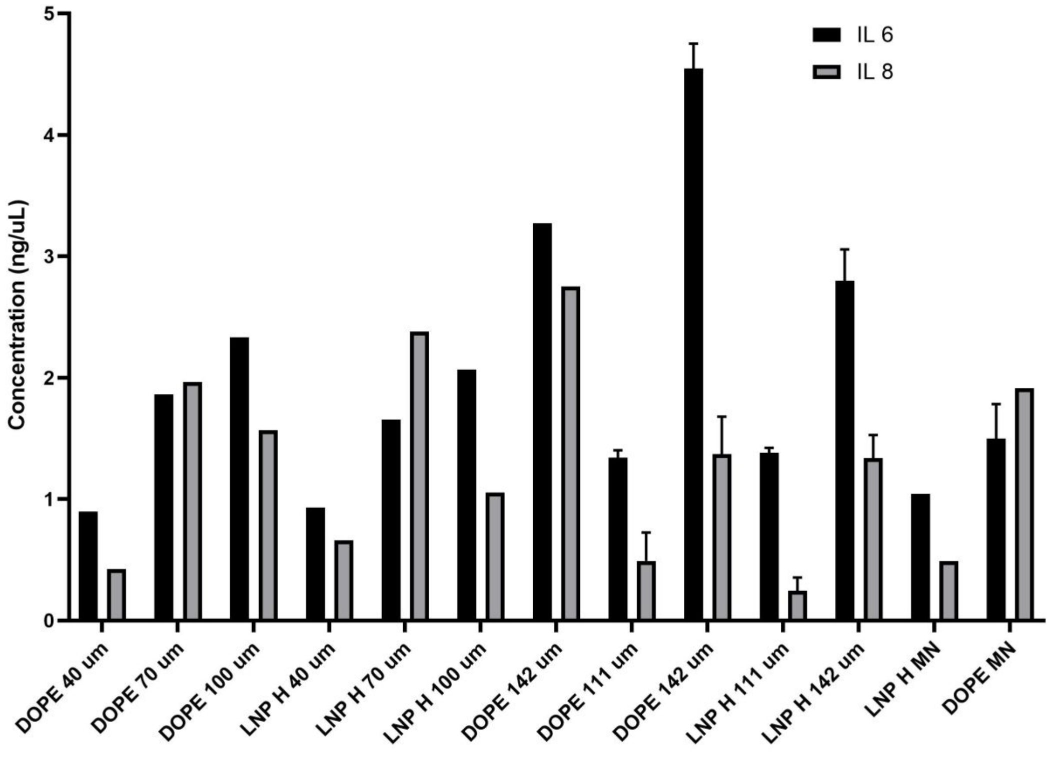

Figure S10. Release of IL6 and IL8 as exemplary makers for skin irritation from freshly excised human skin after pre-treatment with either laser microablation yielding pore formation in different skin depths or 400 µm microneedles (MN) prior the application of DOPE-based LNPs or LNP H.

Assay		LNP H mRNA_donor 1		LNP H mRNA_donor 2			Guide Coordinates				
		Reads	% Indel	Reads	% Indel	Mismatch	Chr	Start	Stop	Strand	ReferenceSequence
HPRT38087_iGS_1.2824	On-Target	10726	**57,81**	7908	**67,62**	0	chrX	134498211	134498231	-	TCCTAGTAATCCCCATAATT
HPRT38087_iGS_2.2713	Off-Target	10541	**0,00**	9893	**0,00**	2	chr4	19711768	19711788	+	CATTATGGGGATT-CTAGGA
HPRT38087_iGS_3.998	Off-Target	51947	**0,00**	51361	**0,04**	4	chr4	84615497	84615517	-	TCCTAGTAGTTCCCTTAATA
HPRT38087_iGS_4.2924	Off-Target	2747	**0,00**	4312	**0,02**	3	chr2	156271175	156271195	-	TCCTAGAAATCCCCATCATC
HPRT38087_iGS_5.672	Off-Target	6057	**0,00**	8360	**0,00**	3	chr18	41320698	41320718	-	CCCTAGTAATCCCCTTAATA
HPRT38087_iGS_6.215	Off-Target	27681	**0,50**	44283	**0,49**	5	chr10	7670462	7670482	+	ATTAGAGGGGACTACTAGGG
HPRT38087_iGS_7.2963	Off-Target	17260	**0,01**	18026	**0,06**	4	chr15	80829814	80829834	+	TTTTATGGGGAGTA-TAGGA
HPRT38087_iGS_8.1896	Off-Target	505	**0,00**	975	**0,00**	5	chrX	64697541	64697561	+	TGTTATAGGGACCACTAGGA
HPRT38087_iGS_9.136	Off-Target	3654	**0,00**	5565	**0,00**	2	chr2	58181373	58181393	+	AATAATGGGGATTTACTAGGA

Assay		Untreated_donor 1		Untreated_donor 2			Guide Coordinates				
		Reads	% Indel	Reads	% Indel	Mismatch	Chr	Start	Stop	Strand	ReferenceSequence
HPRT38087_iGS_1.2824	On-Target	15882	**0,01**	14738	**0,00**	0	chrX	134498211	134498231	-	TCCTAGTAATCCCCATAATT
HPRT38087_iGS_2.2713	Off-Target	11579	**0,01**	11310	**0,00**	2	chr4	19711768	19711788	+	CATTATGGGGATT-CTAGGA
HPRT38087_iGS_3.998	Off-Target	53186	**0,03**	50923	**0,03**	4	chr4	84615497	84615517	-	TCCTAGTAGTTCCCTTAATA
HPRT38087_iGS_4.2924	Off-Target	4630	**0,00**	6222	**0,00**	3	chr2	156271175	156271195	-	TCCTAGAAATCCCCATCATC
HPRT38087_iGS_5.672	Off-Target	11330	**0,00**	11986	**0,00**	3	chr18	41320698	41320718	-	CCCTAGTAATCCCCTTAATA
HPRT38087_iGS_6.215	Off-Target	18301	**0,44**	24171	**0,46**	5	chr10	7670462	7670482	+	ATTAGAGGGGACTACTAGGG
HPRT38087_iGS_7.2963	Off-Target	20725	**0,00**	22714	**0,00**	4	chr15	80829814	80829834	+	TTTTATGGGGAGTA-TAGGA
HPRT38087_iGS_8.1896	Off-Target	1562	**0,00**	2079	**0,05**	5	chrX	64697541	64697561	+	TGTTATAGGGACCACTAGGA
HPRT38087_iGS_9.136	Off-Target	7354	**0,01**	9015	**0,00**	2	chr2	58181373	58181393	+	AATAATGGGGATTTACTAGGA

Figure S11. rhAMP-Sequencing results of primary human keratinocytes after treatment with Cas9 mRNA-loaded LNP H. This table shows the relevant on-target and eight predicted off-target sites and corresponding sequences, the number of reads per site and the indel frequencies in percentage.

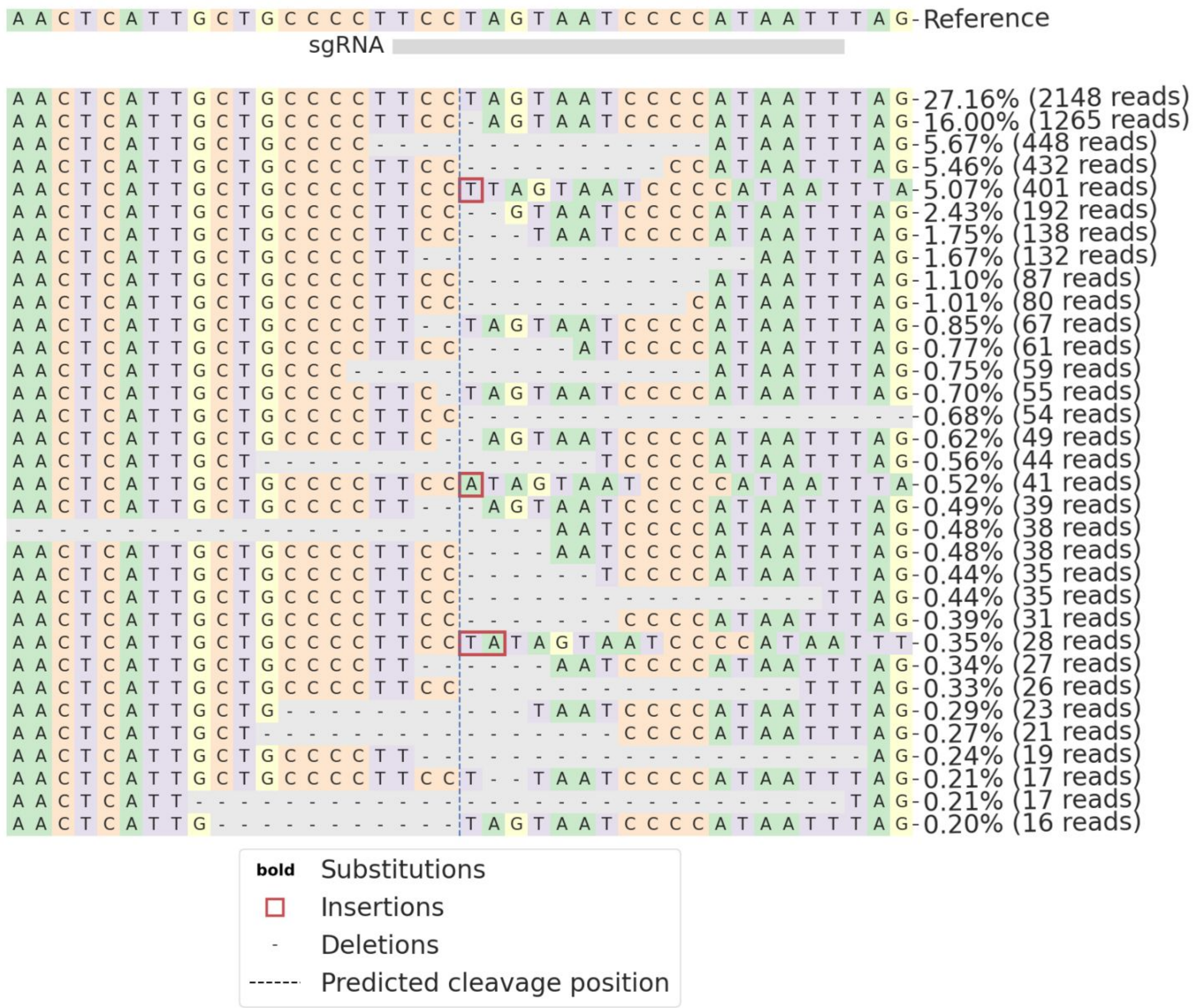

Figure S12. Visualization of the distribution of the most frequently identified alleles around the cleavage site for the sgRNA AATTATGGGGATTACTAGGA in donor 2. Nucleotides are indicated by unique colors (A = green; C = red; G = yellow; T = purple). Substitutions are shown in bold font. Red rectangles highlight inserted sequences. Horizontal dashed lines indicate deleted sequences. The vertical dashed line indicates the predicted cleavage site.

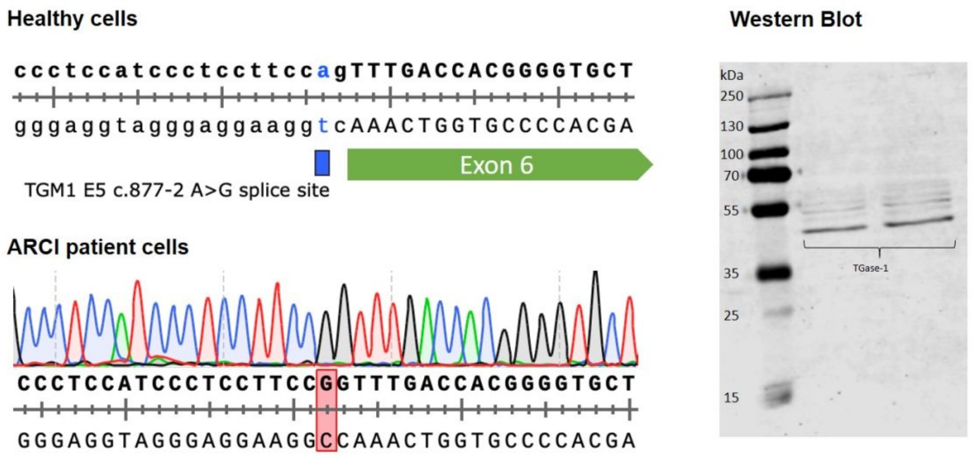

Figure S13. ARCI patient-derived cells were characterized by Sanger sequencing and Western blot clearly showing the mutation at c.877-2 A>G splice site. This mutation causes the expression of a truncated transglutaminase 1 protein which was confirmed by Western blot showing bands at 50 kDa while the full-length protein has a molecular weight of 92 kDa.
